# Microglia-driven inflammation induces progressive tauopathies and synucleinopathies

**DOI:** 10.1038/s12276-025-01450-z

**Published:** 2025-05-01

**Authors:** Sang Hwan Lee, Eun-Jin Bae, Sung Jun Park, Seung-Jae Lee

**Affiliations:** 1 Department of Biomedical Sciences, Seoul National University College of Medicine, Seoul, Republic of Korea; 2 Neuroscience Research Institute, Medical Research Center, Seoul National University, Seoul, Republic of Korea; 3 Convergence Research Center for Dementia, Medical Research Center, Seoul National University, Seoul, Republic of Korea

**Keywords:** Mechanisms of disease, Alzheimer's disease, Parkinson's disease, Neurodegeneration

## Abstract

Alzheimer’s disease and Parkinson’s disease are characterized by distinct types of abnormal protein aggregates within neurons. These aggregates are known as neurofibrillary tangles and Lewy bodies, which consist of tau and α-synuclein, respectively. As the diseases progress, these aggregates spread from one cell to another, causing protein pathology to affect broader regions of the brain. Another notable characteristic of these diseases is neuroinflammation, which occurs when microglia become activated. Recent studies have suggested that inflammation may contribute to the formation and propagation of protein aggregates. However, it remains unclear whether microglia-driven inflammation can initiate and propagate different proteinopathies and associated neuropathology in neurodegenerative diseases. Here, using single-cell RNA sequencing, we observed that microglia exposed to α-synuclein or tau underwent changes in their characteristics and displayed distinct types of inflammatory response. The naive mice that received these microglial cell transplants developed both tauopathy and synucleinopathy, along with gliosis and inflammation. Importantly, these pathological features were not limited to the injection sites but also spread to other regions of the brain, including the opposite hemisphere. In conjunction with these pathological changes, the mice experienced progressive motor and cognitive deficits. These findings conclusively demonstrate that microglia-driven inflammation alone can trigger the full range of pathological features observed in neurodegenerative diseases, and that inflammation-induced local neuropathology can spread to larger brain regions. Consequently, these results suggest that microglia-driven inflammation plays an early and pivotal role in the development of neurodegenerative diseases.

**Figure:**
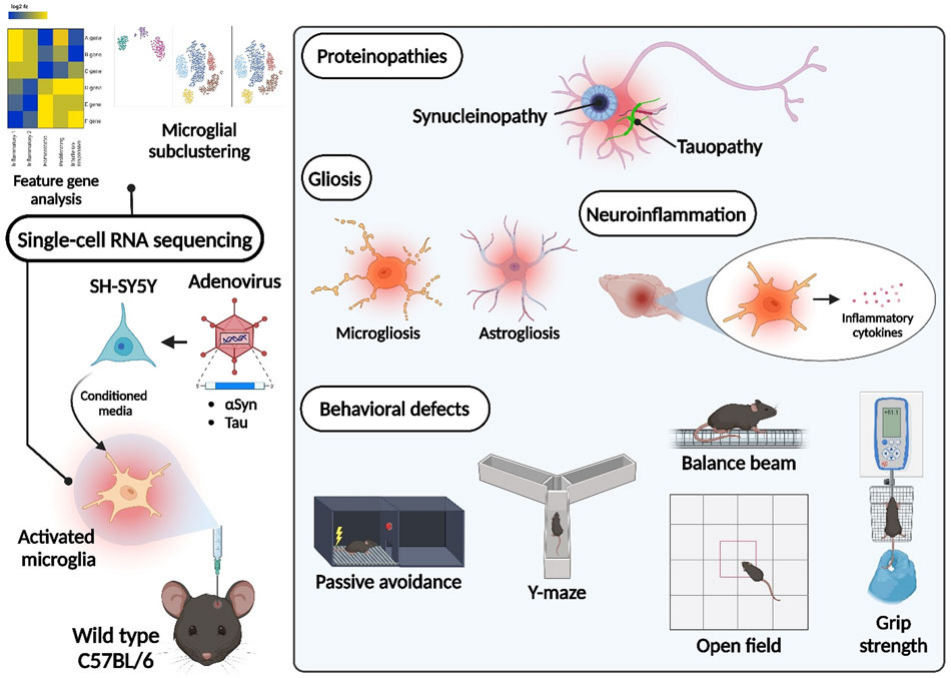
The transplantation of microglia activated by αSyn or tau proteins into the brains of naive mice resulted in the formation of synucleinopathy, tauopathy, gliosis, neuroinflammation and behavioral abnormalities. Activated microglia displayed alterations in subclusters as well as the corresponding feature genes.

The transplantation of microglia activated by αSyn or tau proteins into the brains of naive mice resulted in the formation of synucleinopathy, tauopathy, gliosis, neuroinflammation and behavioral abnormalities. Activated microglia displayed alterations in subclusters as well as the corresponding feature genes.

## Microglia activation initiates tau and α-synuclein spread

Alzheimer’s disease (AD) and Parkinson’s disease (PD) are common brain disorders that cause memory and movement problems. Both diseases involve harmful protein buildups in the brain. Researchers explored how inflammation in the brain might contribute to these diseases. They focused on microglia, which are immune cells in the brain that can cause inflammation. In their study, they activated microglia using proteins linked to AD and PD. These activated microglia were then injected into mice brains. The study found that these microglia caused signs of brain damage like those seen in AD and PD, including protein buildups and inflammation. The researchers used various methods, including single-cell RNA sequencing, to analyze changes in the microglia. The results suggest that inflammation driven by microglia might play a key role in the early stages of neurodegenerative diseases.

This summary was initially drafted using artificial intelligence, then revised and fact-checked by the author.

## Introduction

Alzheimer’s disease (AD) and Parkinson’s disease (PD) are the two most prevalent neurodegenerative diseases that impose substantial social burdens^1^. AD is characterized by the deposition of amyloid plaques and neurofibrillary tangles composed of amyloid β (Aβ) peptide and hyperphosphorylated tau proteins, respectively^2^. PD, meanwhile, is marked by the aggregation of α-synuclein (αSyn) proteins in Lewy bodies (LBs)^3^. Despite their differences in the proteins involved and where the pathology occurs, these disorders share many similarities, suggesting a converging pathophysiology^4^. For example, high levels of αSyn proteins have been observed in the brain and cerebrospinal fluid (CSF) of patients with AD, and amyloid plaques and tau aggregates have been found in the brains of some patients with PD^5,6^, as well as in patients with dementia with LBs^7^. Recent studies have also shown a notable correlation between synucleinopathy and tauopathy^8,9^.

Neuroinflammation has been suggested as a crucial factor contributing to the development of neurodegenerative diseases^10^. Among many brain cell types and some infiltrating blood cells, microglia are primarily known to mediate various neuroinflammatory pathways^11^. Microglia actively phagocytose synaptic structures of neuronal cells and have been implicated in synapse loss in both PD and AD cases^12,13^. In addition, irregular activation of microglia can contribute to metabolic dysfunction and oxidative stress, both of which are strongly associated with neurodegeneration^14,15^. In AD models, depleting microglial cells by pharmacologically inhibiting CSF1R reduces pro-inflammatory signaling and rescues tau pathology, while also protecting against neuroinflammation and dopaminergic neurodegeneration in PD models^16,17^. Furthermore, CSF1R signaling has been highlighted as a regulator of other neurodegenerative diseases, such as multiple sclerosis and spinal muscular atrophy, indicating that microglia perform important functions in a broad spectrum of neurodegenerative disorders^18–21^.

Neuroinflammation may play a critical role in the progression of proteinopathies. In a recent study, we demonstrated that the extent of synucleinopathy lesions correlated with levels of neuroinflammation, and these lesions improved when anti-inflammatory agents were administered in a mouse model^22^. Inflammatory factors such as TNFα and ASC were found to promote the aggregation of αSyn and tau proteins, respectively^23–25^. However, it is still uncertain whether neuroinflammation, particularly microglia-driven neuroinflammation, plays a primary or secondary role. In this study, we activated primary microglia using conditioned media (CM) from SH-SY5Y neuroblastoma that overexpressed and secreted either αSyn or tau proteins. When these microglia were injected into the unilateral striatum of naive mice, they exhibited all the characteristic signs of neurodegeneration, including gliosis, propagation of phosphorylated αSyn and tau proteins, and impairment in motor and cognitive functions. Analysis of single-cell RNA sequencing (scRNA-seq) data revealed significant alterations in factors associated with neuroinflammation in activated microglia. Our findings suggest that neuroinflammatory pathways driven by activated microglia may be an early mechanism underlying various neurodegenerative diseases.

## Materials and methods

### Primary microglial culture

As previously described, primary cells were isolated using trypsin (Sigma, #T8003) from the cerebral cortex of P1 neonatal C57BL/6 mice^26^. After treating with DNase I (Roche, #11284932001), cells were centrifuged at 500*g* for 5 min and then resuspended in DMEM–F12 medium supplemented with 10% fetal bovine serum. Subsequently, cells were filtered and plated onto dishes coated with poly-d-lysine. After 10 days of incubation, primary microglia were isolated by tapping the dishes and then plated onto poly-d-lysine-coated plates.

To prepare activated microglia for stereotactic injection, SH-SY5Y neuroblastoma cells were infected with adenovirus expressing the LacZ, αSyn or tau (4R) proteins. After 18–24 h of infection at 37 °C, cells were incubated with normal growth media for 24 h, followed by incubation with serum-free DMEM for 18 h. After the serum starvation, cell media were collected and centrifuged at 1,000 rpm for 10 min. The supernatants were then centrifuged at 10,000*g* for 20 min to generate CM. Before use, αSyn-CM was diluted to 200 ng/μl of αSyn as measured by enzyme-linked immunosorbent assay. The same dilution factor was applied to LacZ-CM and tau-CM. Next, primary microglia were treated with CM for 2 h at 37 °C. After activation, microglia were detached and resuspended in PBS at a concentration of 2 × 10^4^ or 5 × 10^4^ cells/2 μl. For an experiment examining the spatial distribution of exogenous microglia, cells were pretreated with 50 nM of quantum dot (QD) solution (Invitrogen, Q25011MP) for 2 h at 37 °C before the CM treatment.

### Reverse-transcription quantitative PCR (RT-qPCR)

Total RNA was isolated from primary microglia using RNeasy Mini Kit (Qiagen, #74106). After synthesizing cDNA using iScript cDNA Synthesis Kit (Bio-Rad, #1708891), qPCR was performed using iTaq Universal SYBR Green Supermix (Bio-Rad, #172-5121). The following primers were used: *TNFα* (forward), 5′-CCTCTTCTCATTCCTGCTTGTGG-3′; *TNFα* (reverse), 5′-GGTGGTTTGTGAGTGTGAGGG-3′; *IL1β* (forward), 5′-ATCCCAAGCAATACCCAAAGAAGAA-3′; *IL1β* (reverse), 5′-GTGAAGTCAATTATGTCCTGACCAC-3′; *GAPDH* (forward), 5′-AGAAGGTCGTGAAGCAGGCATC-3′; *GAPDH* (reverse), 5′-CGAAGGTGGAAGAGTGGGAGTTG-3′. Forty cycles (95 °C for 5 s and 58 °C for 30 s each) of the thermocycling program were run on a CFX Connect Real-Time PCR System (Bio-Rad, #1855201). *GAPDH* was used as a reference gene, and ΔΔCt values were used to calculate the relative mRNA levels.

### scRNA-seq

The Chromium Next GEM Single Cell 3p RNA library v3.1 was used according to the manufacturer’s protocol. Libraries were pooled and sequenced on a NextSeq or NovaSeq6000. The sequencing reads were demultiplexed and aligned to the mouse mm10 (3.0.0) reference genome using CellRanger 3.0.2 by 10x Genomics. Cells that did not meet the quality control thresholds were excluded. These thresholds included having fewer than 200 genes, more than 5% mitochondrial content, an excessively high number of identified genes, or unique molecular identifiers. After the quality control procedure, a total of 33,275 single-cell expression profiles were obtained.

### Cell clustering and subcluster analysis

The data integration process was carried out following the instructions provided by Seurat. Variable genes were determined using the method SelectIntegrationFeatures (nfeatures = 3000). The top 3000 variable genes were used for both integration feature selection and integration anchor identification. Subsequently, clusters were identified through the FindNeighbors function with dimensions ranging from 1 to 10, and the FindClusters function with a resolution of 0.5. Marker genes were identified using the FindAllMarkers function with parameters specified as min.pct = 0.25 and log2fc.threshold = 0.25. The identification of marker genes was performed using a significance threshold of *P* < 0.05. According to the Panglao database, oligodendrocytes (identified by MBP and PLP1), astrocytes (AQP4 and GFAP) and microglia (CSF1R and AIF1) were annotated. The subset function was used to isolate microglia clusters (LacZ-Mg = 9,929, αSyn-Mg = 10,582 and tau-Mg = 9,565 cells) from the original Seurat object. Using the FindAllMarkers method with parameters of min.pct = 0.25 and log2fc.threshold = 0.3, marker genes were identified through data integration and unsupervised clustering at a resolution of 0.5. Significantly differentially expressed genes (DEGs) were those that displayed a log_2_ fold change greater than 0.3 or less than −0.3, and a Benjamini–Hochberg-corrected *P* value below 0.05. For the enrichment analysis of Gene Ontology (GO) terms, we used the Cytoscape plug-in ClueGO, considering statistical significance and associated terms.

### 2′-7′Dichlorofluoresceine assay

To measure levels of reactive oxygen species (ROS), microglia were treated with 50 μM of cell permeable dye, 2′-7′-dichlorodihydrofluoresceine diacetate (H2DCFDA) for 15 min. Fluorescence was then detected using Synergy Neo plate reader (BioTek).

### Mice

Nine-week-old C57BL/6 male mice were purchased from Orient Bio. Mice were fed ad libitum and habituated for 2 weeks before microglial transplantation. All animal experiments complied with the regulations of the Seoul National University Institutional Animal Care and Use Committee.

### Stereotactic injection

Eleven-week-old C57BL/6 male mice were anesthetized using mixtures of ketamine hydrochloride and xylazine hydrochloride (3.5:1, 2.5 ml/kg). After applying ointment to prevent the eyes from drying out, the head of the mouse was secured using a stereotactic apparatus. A small incision was then made on the skin, and the right striatum (mediolateral [ML], −1.5; anteroposterior [AP], 1.0; and dorsoventral [DV], −3.0 mm) was targeted using bregma as a reference point. After making a small hole on the skull, 2 μl of primary microglia (2 × 10^4^ or 5 × 10^4^ cells) were injected into the unilateral striatum using a Hamilton syringe (Hamilton, 80030) at a rate of 0.5 μl/min. The needle was held in place for 2 min to prevent any backflow, and then retracted at a rate of 1.8 mm/min.

### Behavioral tests

The motor functions of mice were evaluated by measuring their balance beam performance and grip strength. In the balance beam test, the mice were placed on a straight beam (100 cm in length, 2 cm in width and 50 cm in height) and observed as they crossed to a destination point. Through video analysis, the number of hindlimb slips was counted. In the grip strength test, a grip strength meter was used to measure the strength of both the forelimbs and hinblimbs.

To assess the nonmotor functions of the mice, the passive avoidance test and Y-maze test were used. In the passive avoidance test, the mice were placed in a dark compartment and subjected to a 1 mA electric shock for 2 s. Mice were then moved to a lit compartment and observed as they moved toward the dark compartment. The time it took for them to enter the dark compartment was automatically recorded using a laser-dependent time recording system. In the Y-maze test, the mice were placed on a Y-shaped maze with three arms, and their entries into each arm were recorded for 7 min. The proportion of spontaneous alternation was then calculated.

For the open field test, the mice were placed in the center of a white box (40 cm in length, 40 cm in width and 40 cm in height). A video tracking system and automated software (Ethovision XT 14) were used to measure the total distance moved and the time spent at the center point.

### Western blot analysis

After dissecting the whole brains of mice, total proteins from the ipsilateral/contralateral striatum, cerebral cortex, and hippocampus were extracted using 1% Triton X-100 (Bio-Rad, #161-0407) lysis buffer containing a Protease/Phosphatase Inhibitor Cocktail (Sigma, P8340 and P0044). After adding the sample buffer, samples were boiled at 99 °C for 10 min. Next, electrophoresis was performed on 8–12% polyacrylamide gels. The proteins were then transferred to nitrocellulose membranes (Whatman, #10600001), which were blocked using a TBST solution containing 5% bovine serum albumin (BSA; Sigma, A7906). After an overnight (O/N) incubation with a primary antibody solution containing 3–5% BSA at 4 °C, the membranes were incubated with a secondary antibody solution containing 3–5% skim milk for 1 h. Finally, blot images were captured using an Amersham Imager 600 (GE Healthcare) after incubation with the HRP substrate solution (Millipore, WBKLS0100) for 1 min. The following primary antibodies were used: anti-αSyn, BD Transduction, #610787; anti-tau (4R), Sigma, #05-804; anti-αSyn (pS129), Abcam, ab51253; anti-p-tau (Ser202, Thr205), Invitrogen, #05-804. The following secondary antibodies were used: goat anti-rabbit IgG-HRP conjugate, Bio-Rad, #1706515; goat anti-mouse IgG-HRP conjugate, Bio-Rad, #1706516.

### Immunohistochemical (IHC) assays

Mice were subjected to cardiac perfusion using a saline solution and a 4% paraformaldehyde (Sigma, P6148) solution. The whole brains of mice were collected and then postfixed with a 4% paraformaldehyde solution at 4 °C O/N. Brain slices of 40 μm were obtained using a vibratome (Leica, VT1200S). After 30 min of incubation with a 3% H_2_O_2_ solution (Sigma, H1009), the samples were incubated for 1 h with PBST solution containing 0.1% Triton X-100 and 4% BSA. Next, the samples were incubated with a primary antibody solution at 4 °C O/N, followed by incubation with a secondary antibody solution for 1 h. After the avidin–biotin complex (ABC) reaction (Vector Laboratories, PK-6200) for 1 h, the samples were incubated with a 0.1 M Tris–HCl solution (Sigma, T3253) containing 3,3′-diaminobenzidine (Sigma, D5637). The samples were visualized using a light microscope (Olympus, SZ51) and placed on a slide glass (Marienfeld, HSU-0810001). The slides were then air-dried O/N at room temperature, followed by washing with ethanol and treatment with xylene (SAMCHUN, X0097). Once the samples were mounted with Canada Balsam (Sigma, C1795), images were captured using a microscope (Zeiss, Axiocam 208 color) and compatible software (Zeiss, Zen 3.6). The following primary antibodies were used: anti-αSyn, BD Transduction, #610787; anti-tau (4R), Sigma, #05-804; anti-αSyn (pS129), Abcam, ab51253; anti-p-tau (Ser202, Thr205), Invitrogen, #05-804; anti-Iba1, FUJIFILM Wako, #019-19741; anti-GFAP, Abcam, ab7260; and anti-TNFα, Novus, NBP1-19532. The following secondary antibody was used: biotinylated horse anti-mouse/rabbit IgG antibody (Universal), Vector Laboratories, PK-6200.

### Immunofluorescence (IF)

The brain slices were incubated with blocking and primary antibody solutions. After being treated with a secondary antibody solution, the samples were mounted on a slide glass using a mounting solution containing DAPI (Vector Laboratories, H-1200). When performing thioflavin S (Thio-S) staining, the samples were incubated with a 50% EtOH/dH_2_O solution containing 0.05% Thio-S (Sigma, T1892) for 8 min, following incubation with a secondary antibody solution for 1 h. To remove any excess fluorochrome, the samples were washed twice with 80% EtOH. IF was observed and images were acquired using a confocal microscope (Zeiss, LSM 980). The following primary antibodies were used: anti-αSyn (pS129), Abcam, ab51253; anti-p-tau (Ser202, Thr205), Invitrogen, #05-804; anti-Iba1, FUJIFILM Wako, #019-19741; anti-Iba1, FUJIFILM Wako, #011-27991; and anti-MAP2, Novus, NBP3-05552. The following secondary antibodies were used: Cy2 donkey anti-mouse IgG, Jackson ImmunoResearch, #715-225-150; RRX Donkey Anti-Rabbit IgG, Jackson ImmunoResearch, #711-295-152; Alexa Fluor 647 donkey anti-goat IgG, Jackson ImmunoResearch, #705-605-147; Alexa Fluor 488 goat anti-mouse IgG, Jackson ImmunoResearch, #115-545-003; Alexa Fluor 647 goat anti-rabbit IgG, Jackson ImmunoResearch, #111-605-144; and Alexa Fluor 488 goat anti-rabbit IgG, Jackson ImmunoResearch, #111-545-144.

### Image analysis

The IHC and IF images were analyzed using the Fiji software (National Institutes of Health). For the IHC images, the color deconvolution function was used for stain separation. After setting specific thresholds for each antibody to exclude background signals, corrected optical density values were obtained. For the IF images, the Cell Counter plugin was used to count the number of cells and immunoreactive puncta. GraphPad Prism was used to create the graphs.

### Research Resource Identifiers (RRID)

Antibodies: anti-αSyn antibody, BD Biosciences, cat. no. 610787, RRID: AB_398108; anti-tau antibody, Millipore, cat. no. 05-804, RRID: AB_310014; anti-αSyn (Ser129) antibody, Abcam, cat. no. ab-51253, RRID: AB_869973; anti-phospho-tau antibody, Thermo Fisher Scientific, cat. no. MN1020, RRID: AB_223647; goat anti-rabbit IgG-HRP conjugate, Bio-Rad, cat. no. 170-6515, RRID: AB_11125142; goat anti-mouse IgG-HRP conjugate, Bio-Rad, cat. no. 1706516, RRID: AB_2921252; anti-Iba1, FUJIFILM Wako Shibayagi, cat. no. 019-19741, RRID: AB_839504; anti-GFAP antibody, Abcam, cat. no. ab7260, RRID: AB_305808; anti-TNFα antibody, Novus, cat. no. NBP1-19532, RRID: AB_2271897; VECTASTAIN Elite ABC-Peroxidase Kit, Vector Laboratories, cat. no. PK-6200, RRID: AB_2336826. Software: Ethovision XT, RRID: SCR_000441; GE Amersham Imager 600, RRID: SCR_021853; Fiji, RRID: SCR_002285; GraphPad, RRID: SCR_000306; Zeiss Microscopy, RRID: SCR_023607.

### Statistical analysis

All values are presented as mean ± standard error of the mean (s.e.m.). Differences between the means of two groups were analyzed using unpaired *t*-tests, while one-way analysis of variance (ANOVA) with Tukey’s post hoc test was used when there were three or more independent groups with one independent variable. For comparing groups with two independent variables, two-way ANOVA followed by Tukey’s post hoc test was performed. In behavioral tests, statistical significance was determined by two-way repeated measures ANOVA followed by Šidák’s post hoc test. *P < 0.05, **P < 0.005, ***P < 0.001, ****P < 0.0001.

## Results

### Gene expression patterns of microglial subtypes altered by αSyn and tau

**Fig. 1:**
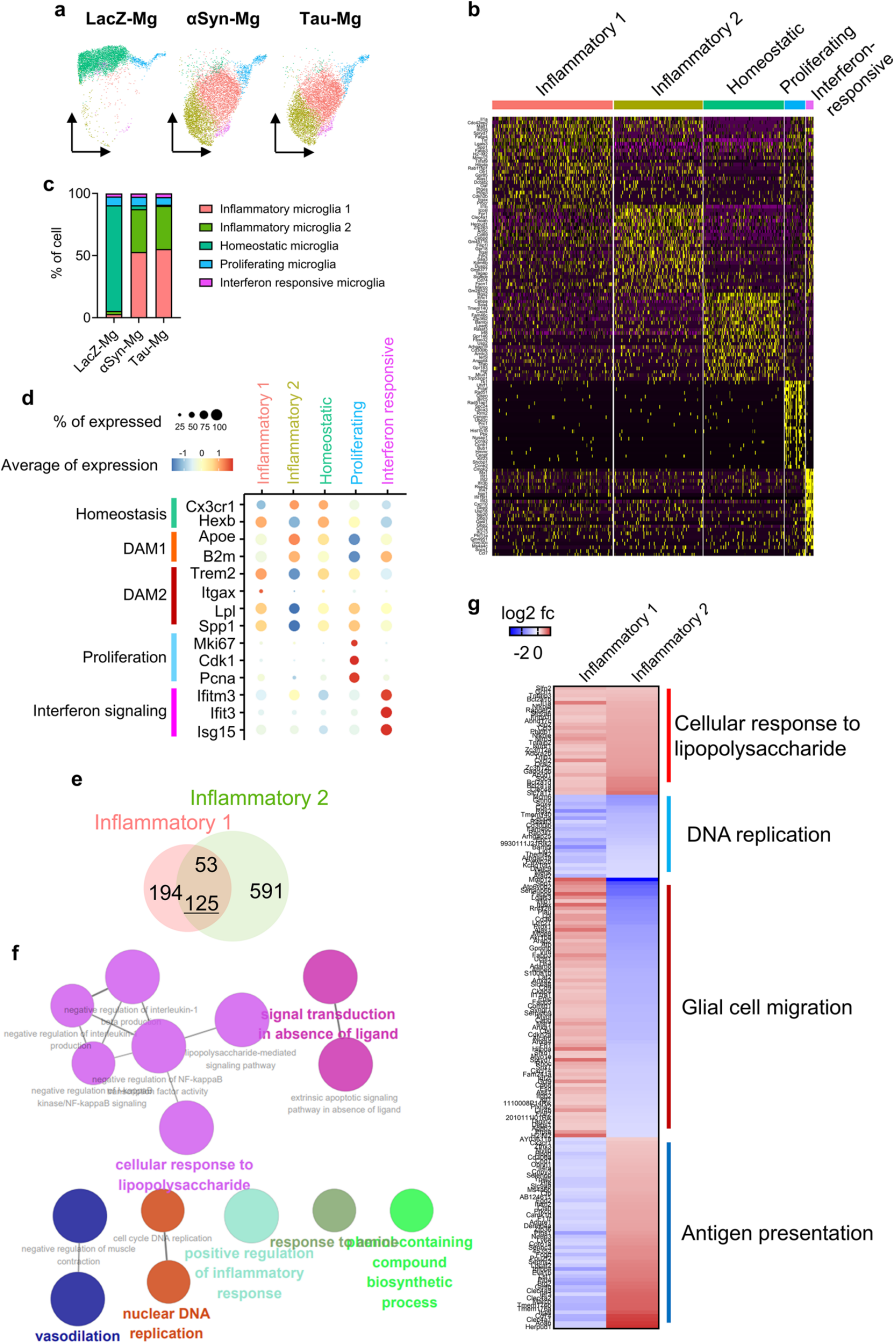
The expression profiles and subtype clustering of microglia change in response to protein aggregates. **a** The UMAP representation of 22,680 cells, showing the separation into five clusters. **b** Heatmaps of the top 100 feature genes from each subcluster shown in **a**. Based on these feature genes, the microglial clusters were defined as inflammatory microglia 1, inflammatory microglia 2, homeostatic microglia, proliferating microglia and interferon-responsive microglia. **c** A stacking bar graph displaying the percentage of cells in each cluster. Treatment of pathogenic protein aggregates reduces the resting and homeostatic populations while increasing the inflammatory microglia. **d** A dot plot showing the expression of relevant marker genes among each microglia cluster from the scRNA-seq dataset shown in **a**. In the dot plot, microglial clusters are visualized in columns and rows represent key functional genes that are differentially expressed in certain clusters. The size of each dot represents the fraction of cells in a given cluster in which the gene was detected, and the color of the dot represents the mean expression *z* score for the cells belonging to that cluster. **e** A Venn diagram comparing the feature genes in inflammatory microglia 1 and 2. The numerical values indicate the number of featured genes, with those showing reversed expression between the groups underlined. **f** The simplified networks of significantly enriched GO terms using 178 overlapping feature genes depicted in **e**. Each term in the network is statistically significant (Benjamini–Hochberg correction <0.05). The nodes (colored circles) represent significantly enriched parent GO terms, and the edges (lines between the nodes) show overlapping genes within terms. The different sizes of the nodes represent the number of enriched genes. **g** Heatmaps of common feature genes in inflammatory microglia 1 and 2. Enriched GO terms were identified by GO enrichment analysis.

To evaluate the effects of neuron-derived αSyn and tau on microglia, we exposed primary microglia from 1-day-old (P1) C57BL/6 mice to CM collected from SH-SY5Y neuroblastoma cells expressing β-galactosidase (LacZ), αSyn or tau proteins. We observed that microglia treated with αSyn-CM or tau-CM displayed significant upregulation of ROS levels and mRNA expression of *TNFα* and *Il1β* (Supplementary Fig. 1a–c). It is well established that microglia, originally recognized as a key regulator of neuroinflammation in various neurodegenerative diseases, consist of distinct subtypes that play specific roles in disease states^27–29^. For instance, microglia expressing CX3CR1 or CD11c have been implicated in AD pathology, while microglial expression of 5D4-KSPG and CCl4 is associated with amyotrophic lateral sclerosis and aging, respectively^30–33^. To understand how the microglial population changes with treatment of αSyn or tau proteins, we performed scRNA-seq and categorized the resulting cells into five subclusters based on their gene expression patterns (Fig. 1a, b). We found that αSyn-CM or tau-CM treatment significantly changed the proportions of homeostatic and inflammatory microglia subclusters 1 and 2. However, the number of proliferating and interferon-responsive microglia remained relatively consistent. In the LacZ-Mg group, 85% of cells were identified as homeostatic microglia, displaying features associated with homeostasis and resting, such as *Cx3cr1*, *Cst3* and *Trem2*. By contrast, only 3% and 0.99% of cells in the αSyn-Mg and tau-Mg groups were classified as homeostatic microglia, respectively^34,35^. Meanwhile, 87.41% and 89.99% of cells in the αSyn-Mg and tau-Mg groups were categorized as inflammatory microglia subclusters, respectively (Fig. 1c).

We aimed to identify the molecular basis of the differences between two major subtypes of microglia, namely inflammatory microglia 1 and 2, in the αSyn-Mg and tau-Mg groups, based on feature genes. We discovered that both subtypes showed high expression of inflammatory genes, including *Il1α*, *Cxcl2* and *Clec4e* (Fig. 1b and Supplementary Table 1). We then proceeded to examine the enrichment of feature genes associated with disease-associated microglia (DAM) to identify molecular characteristics within the different microglial subpopulations. The genes that make up the DAM signature were found to be enriched exclusively in both inflammatory microglia 1 and 2, with each exhibiting a unique combination of DAM1 and DAM2 enrichment. Specifically, inflammatory microglia 1 showed a considerable enrichment for DAM2 signatures and genes implicated in inflammasome pathways, such as *Nlrp3*, *Il1β* and *Ctsb*, suggesting activation of the NLRP3 inflammasome pathway in that particular subgroup of microglia (Fig. 1d and Supplementary Table 1). Although there was an overlap of 178 feature genes in inflammatory microglia 1 and 2, only 53 of these feature genes displayed similar expression patterns in these two distinct subpopulations (Fig. 1e).

These observations prompted us to study the GO enrichment based on the expression of specific feature genes in inflammatory microglia 1 and 2. The 53 genes shared by inflammatory microglia 1 and 2 were significantly enriched in GO terms associated with nuclear DNA replication and positive regulation of inflammatory responses (Fig. 1f, g). Inflammatory microglia 1, based on its unique feature genes, showed a stronger association with lipid metabolism and immune cell migration, while inflammatory microglia 2 was closely related to the activation of toll-like receptor signaling pathways, antigen presentation and processing, and immune cell migration (Supplementary Figs. 2 and 3 and Supplementary Table 2). No significant differences were observed between the αSyn-Mg and tau-Mg groups, as demonstrated by the comparison of expression levels for the top 90 feature genes (Supplementary Fig. 4). It is possible that inflammatory microglia 2 may be responsible for inducing immune responses in neurodegenerative diseases, as aggregate forms of tau and αSyn act as endogenous ligands for microglial TLR2 and cause neurotoxicity in a TLR2-dependent manner^36–38^. Overall, our data suggest that exposure to pathological protein aggregates increases the expression of genes related to inflammatory reactions. These findings highlight the significant differences in molecular features among microglial subpopulations induced by αSyn and tau, and imply that the transition of microglia into unique subtypes with increased inflammatory and metabolic phenotypes may play an important role in the induction of immune responses and proteinopathies in neurodegenerative diseases.

### Spatiotemporal distribution of activated microglia after transplantation into the striatum of naive mice

To determine the optimal number of microglia to inject, we injected either 2 × 10^4^ (low) or 5 × 10^4^ (high) microglia into the unilateral striatum of mice. Using an antibody specific for Iba1, a marker for microglia, we observed that the size of the microglial cell bodies and the ratio of cell body to cell size were increased near the injection site in the αSyn-Mg and tau-Mg groups (Supplementary Fig. 5a–c). These parameters were not increased in the LacZ-Mg-low group. However, the LacZ-Mg-high group showed some changes in microglia compared with the LacZ-Mg-low group. Therefore, we chose to inject the low number (2 × 10^4^) of microglia throughout this study. This number of cells was sufficient to maintain increased microgliosis near the injection site for up to 3 months (Supplementary Fig. 6a–f).

**Fig. 2:**
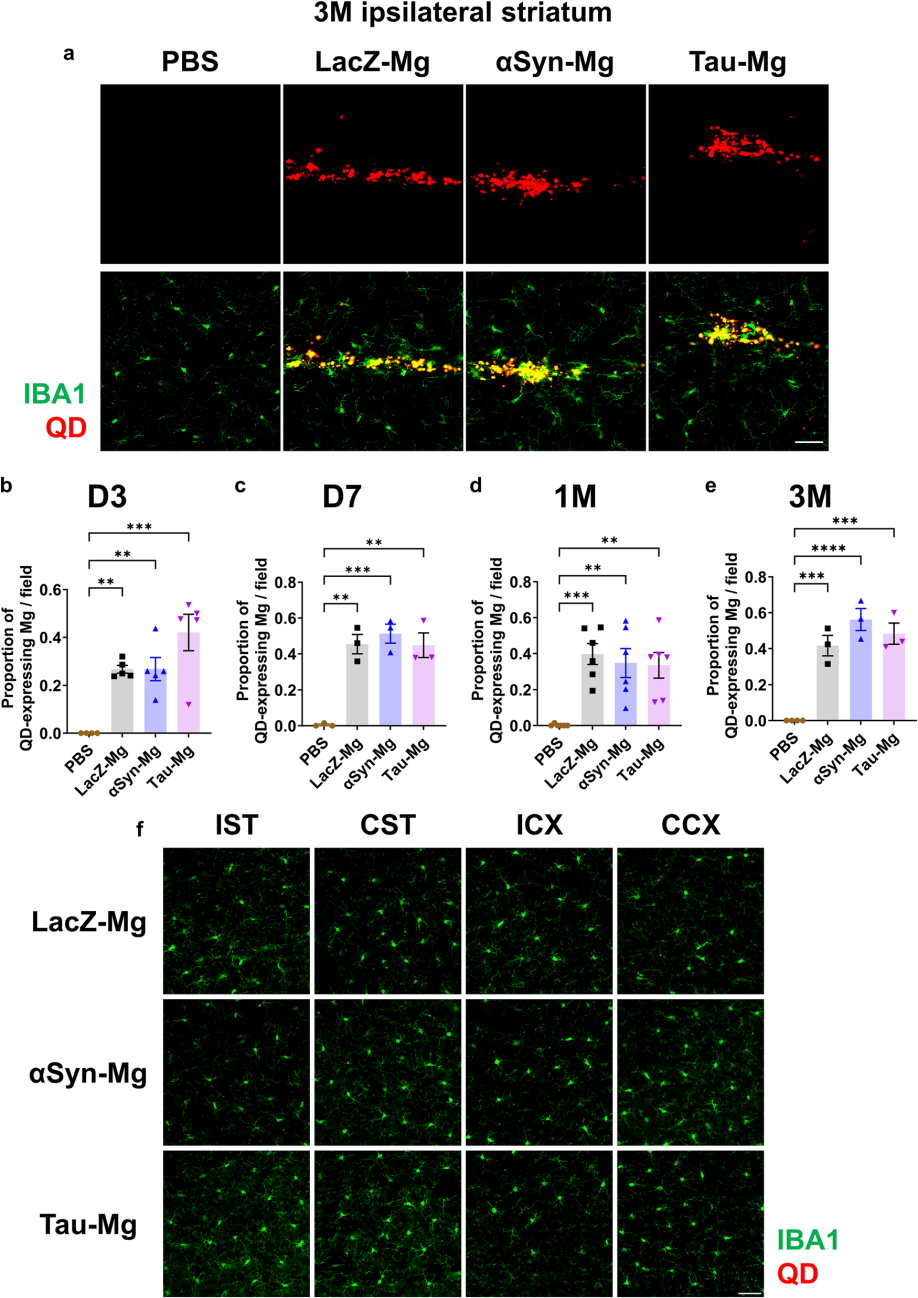
QD-expressing microglia exhibit local distribution near the injection sites. **a** Representative IF images of the ipsilateral striatum labeled with an antibody specific for Iba1 3 months after injection of activated microglia pretreated with QDs. Scale bar, 30 μm. **b**–**e** The proportion of QD-expressing microglia in the ipsilateral striatum 3 days (**b**), 7 days (**c**), 1 month (**d**) and 3 months (**e**) after injection. All data are presented as the mean ± s.e.m. Statistical significance was determined by one-way ANOVA with Tukey’s post hoc test. **f** Representative IF images of the brain regions labeled with an antibody specific for Iba1 3 months after injection of activated microglia pretreated with QDs. Scale bar, 50 μm. IST, ipsilateral striatum distal to injection area; CST, contralateral striatum; ICX, ipsilateral motor cortex; CCX, contralateral motor cortex.

To examine the distribution of the introduced microglia, the cells were pretreated with QDs before being injected into the striatum of mice. The whole brain of the mice was collected at 3 days, 7 days, 1 month and 3 months after injection, and then subjected to a fluorescence imaging. The QD signals were detected only along the needle tract at all the examined time points (Fig. 2a–e and Supplementary Fig. 7). No QD signal was detected in other brain regions distant from the injection site (Fig. 2f and Supplementary Fig. 8). Furthermore, we costained QDs with human forms of αSyn or tau at 3 months post-injection and found that both αSyn and tau proteins were exclusively located within the QD-positive microglial cells (Supplementary Fig. 9).

### Effects of activated microglia on the phosphorylation and propagation of the αSyn and tau proteinsfraction of the αSyn-Mg group (Fig. 3a,b andSupplementary Figs. 10 and 11). We

**Fig. 3:**
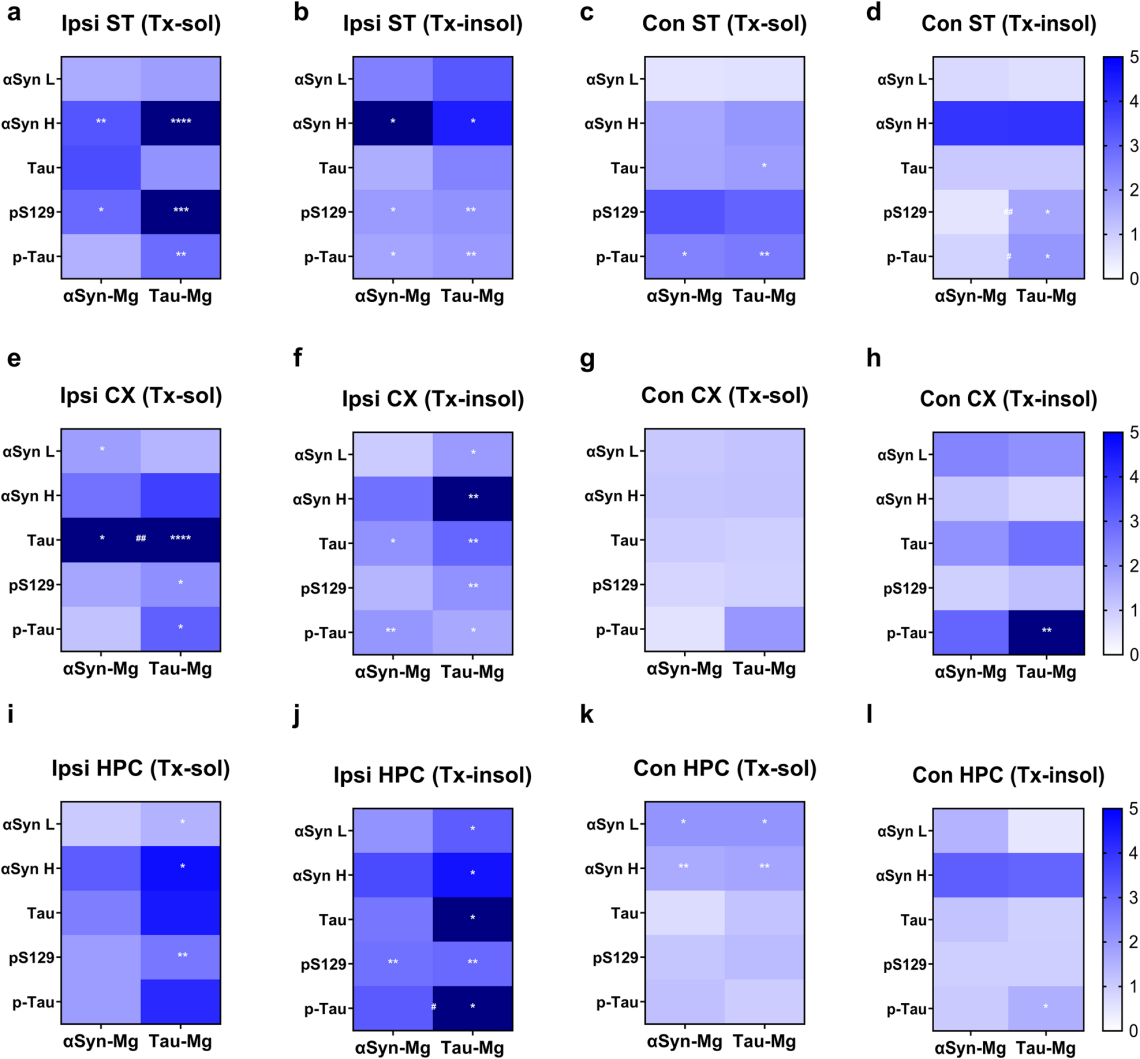
Activated microglia induce phosphorylation and propagation of the αSyn and tau proteins. **a**–**l**, Heatmaps showing the expression patterns of the αSyn, tau, p-αSyn (pS129) and p-tau proteins in the striatum (**a**–**d** respectively), cerebral cortex (**e**–**h**, respectively) and hippocampus (**i**–**l**, respectively). For each value, the relative expression levels of the αSyn-Mg and tau-Mg groups were colored as shown on the right after normalizing to the LacZ-Mg group. For statistical analysis, one-way ANOVA with Tukey’s post hoc test was performed as shown in Supplementary Figs. 6–17. Statistical significance is indicated with white asterisks. *LacZ-Mg versus αSyn-Mg or tau-Mg; ^#^αSyn-Mg versus tau-Mg. αSyn L indicates 19 kDa, and αSyn H indicates molecular weights higher than 19 kDa. CX, cerebral cortex; HPC, hippocampus.

To investigate whether microglia activated by αSyn or tau could cause proteinopathies, we extracted total proteins from the ipsilateral/contralateral striatum, cerebral cortex and hippocampus of mice 4 weeks after injection. The extracted proteins were then subjected to western blot analysis using antibodies specific for αSyn, tau, phosphorylated-αSyn (pS129) and phosphorylated-tau (p-tau) proteins (Fig. 3 and Supplementary Figs. 10–21). In both the detergent-soluble (Tx-sol) and detergent-insoluble (Tx-insol) fractions of the ipsilateral striatum, we observed a significant increase in the levels of high-molecular-weight oligomeric αSyn and pS129 proteins in the αSyn-Mg and tau-Mg groups, as well as an increase in p-tau levels in both fractions of the tau-Mg group and in the Tx-insol fraction of the αSyn-Mg group (Fig. 3a, b and Supplementary Figs. 10 and 11). We also found an overall increase in protein expression, including tau, pS129 and p-tau, in both fractions of the contralateral striatum (Fig. 3c, d and Supplementary Figs. 12 and 13). Importantly, we observed similar upregulation patterns in all fractions of the cortex and hippocampus, indicating that the increase in protein levels was not limited to the striatum (Fig. 3 and Supplementary Figs. 14–21).

**Fig. 4:**
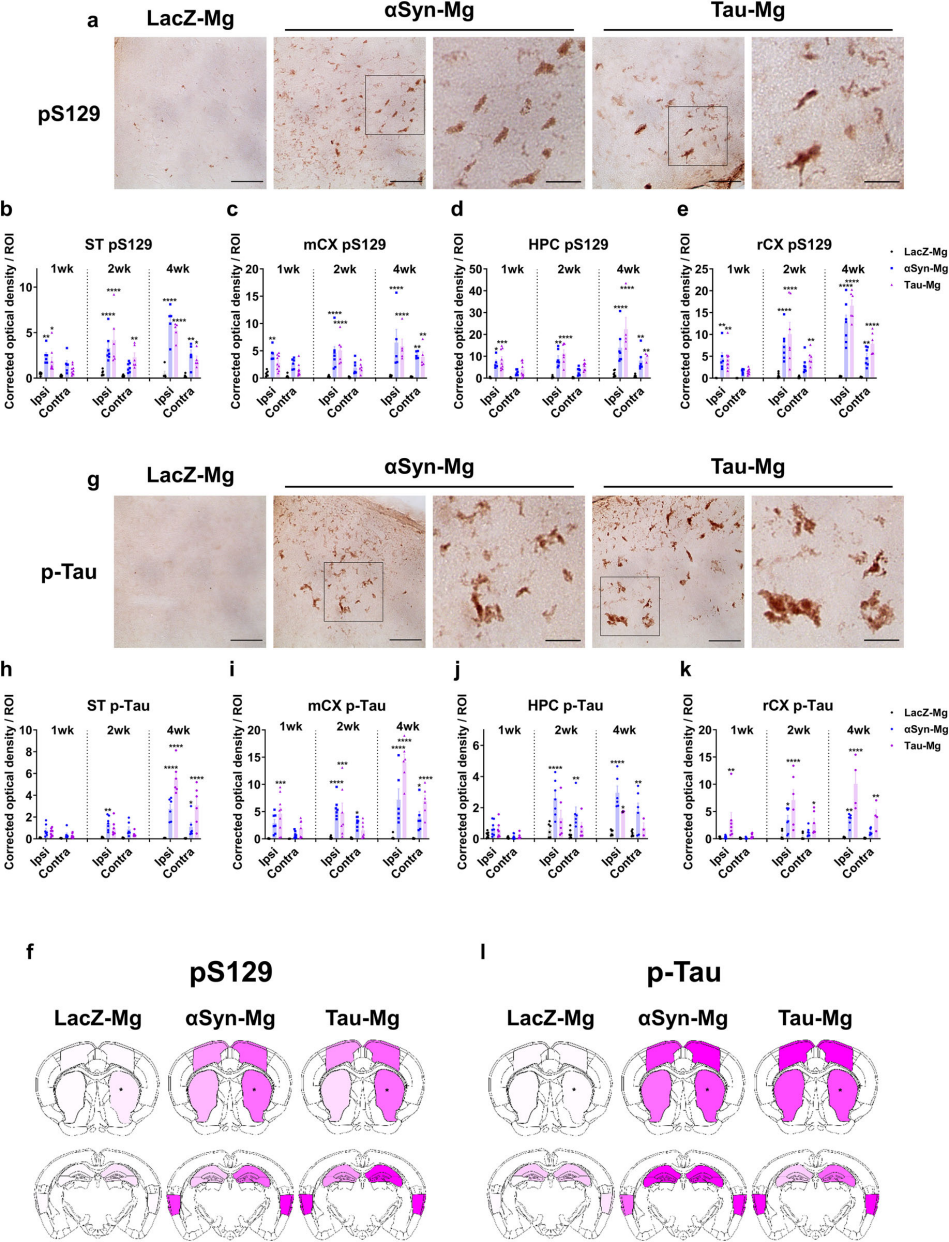
Synucleinopathy and tauopathy are found in the extensive brain regions after microglial transplantation. **a** Representative IHC images of the motor cortex labeled with an antibody specific for pS129 4 weeks after injection. The region of interest (ROI) shown in the black box is magnified. Scale bars, 50 μm and 20 μm (for magnified images). **b**–**e** The relative optical density of pS129 in the striatum (**b**), motor cortex (**c**), hippocampus (**d**) and rhinal cortex (**e**) 1, 2 and 4 weeks after injection. All data are presented as the mean ± s.e.m. For statistical analysis, two-way ANOVA followed by Tukey’s post hoc test was performed. **f**, Heatmaps depicting the expression patterns of pS129. Asterisks mark the injection sites. **g** Representative IHC images of the motor cortex labeled with an antibody specific for p-tau 4 weeks after injection. The ROI shown in the black box is magnified. Scale bars, 50 μm and 20 μm (for magnified images). **h**–**k** The relative optical density of p-tau in the striatum (**h**), motor cortex (**i**), hippocampus (**j**) and rhinal cortex (**k**) 1, 2 and 4 weeks after injection. All data are presented as the mean ± s.e.m. For statistical analysis, two-way ANOVA followed by Tukey’s post hoc test was performed. **l** Heatmaps depicting the expression patterns of p-tau. Asterisks mark the injection sites.

Inspired by the finding that the levels of pS129 and p-tau proteins were increased in extensive brain regions distant from the injection site, we set out to investigate the development and spread of these protein lesions in different brain regions. In our IHC assays, we observed elevated relative optical density (OD) values of pS129 and p-tau as early as 1 week after injection. These values gradually increased over time in all the brain regions we examined (Fig. 4a–l and Supplementary Fig. 22). There were no noticeable differences in the spreading patterns of pS129 and p-tau between the αSyn-Mg and tau-Mg groups. At 4 weeks post-injection, both pS129 and p-tau levels were significantly elevated in tyrosine hydroxylase (TH)-positive dopaminergic neurons within the substantia nigra (SN) in the αSyn-Mg and tau-Mg groups (Supplementary Fig. 23). We subsequently assessed whether other proteinopathies also occured in our injection model and found that Aβ pathology was not detected in the hippocampus and rhinal cortex (rCX) of mice injected with αSyn-Mg or tau-Mg (Supplementary Fig. 24a–c). Moreover, neuronal TDP43 expression did not show significant differences between the groups (Supplementary Fig. 24d–f).

**Fig. 5:**
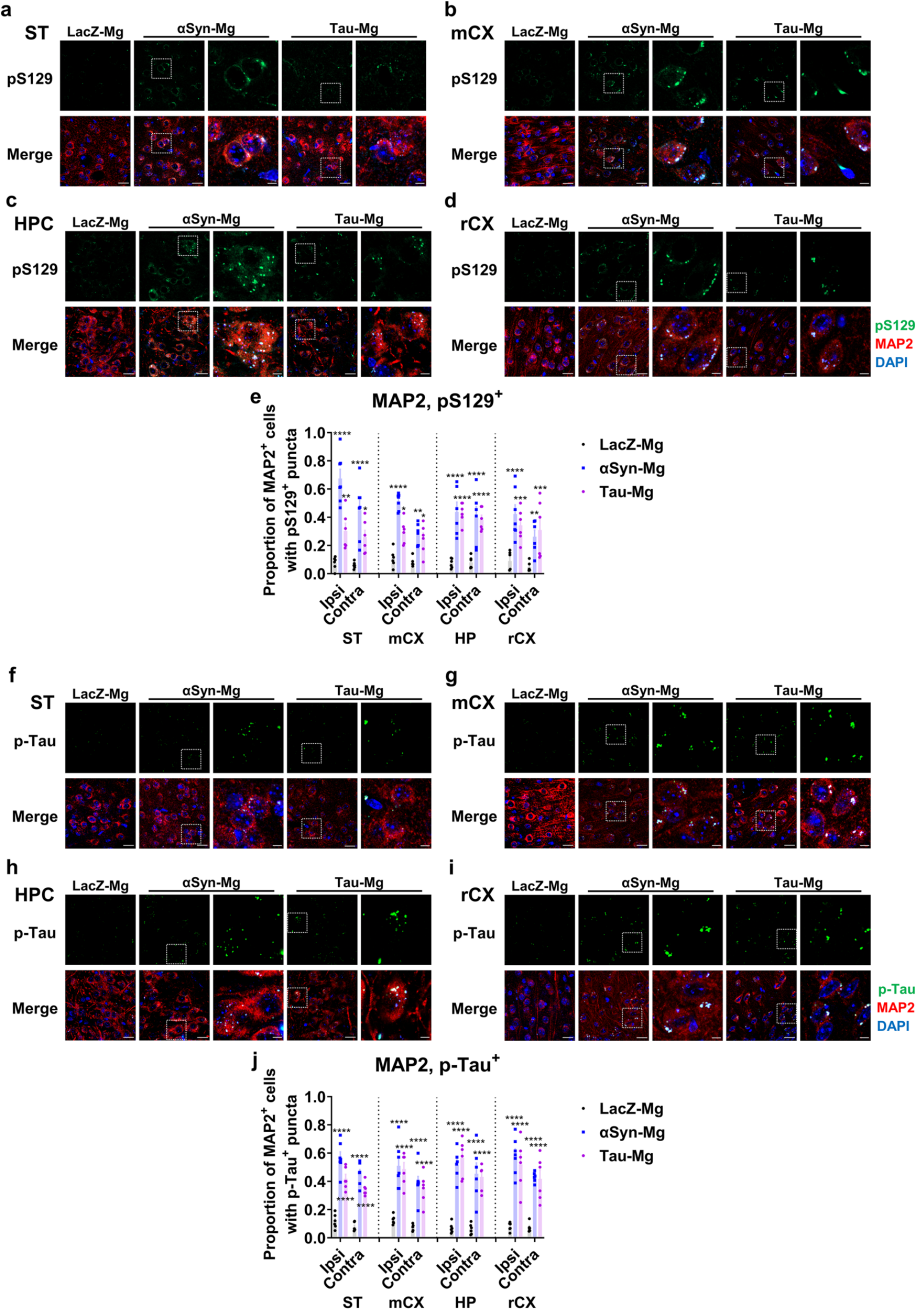
pS129 and p-tau inclusions are enriched in neuronal cells. **a**–**d** Representative IF images of the ipsilateral striatum (**a**), motor cortex (**b**), hippocampus (**c**) and rhinal cortex (**d**) costained with antibodies specific for MAP2 and pS129 1 month after injection. The ROI shown in the white box is magnified. Scale bars, 20 μm and 10 μm (for magnified images). **e** The proportion of MAP2^+^ cells with pS129^+^ inclusions. All data are presented as the mean ± s.e.m. For statistical analysis, two-way ANOVA followed by Tukey’s post hoc test was performed. **f**–**i** Representative IF images of the ipsilateral striatum (**f**), motor cortex (**g**), hippocampus (**h**) and rhinal cortex (**i**) costained with antibodies specific for MAP2 and p-tau 1 month after injection. The ROI shown in the white box is magnified. Scale bars, 20 μm and 10 μm (for magnified images). **j** The proportion of MAP2^+^ cells with p-tau^+^ inclusions. All data are presented as the mean ± s.e.m. For statistical analysis, two-way ANOVA followed by Tukey’s post hoc test was performed.

Next, we conducted costaining of pS129 and p-tau with MAP2 and Iba1, markers for neurons and microglia, respectively, to determine the cellular source of pS129 and p-tau expression. In the αSyn-Mg and tau-Mg groups, we observed a significant increase in the colocalization of MAP2-positive neurons with pS129 and p-tau in various regions, including the striatum, motor cortex, rhinal cortex and hippocampus (Fig. 5a–j). In addition, we observed intracellular deposition of pS129 and p-tau in Iba1-positive microglia, albeit with a comparatively lower accumulation of pS129 (Supplementary Figs. 25 and 26). We then measured the number of pS129 and p-tau puncta in cells that were positive for Iba1 or MAP2, and observed a significant increase in both neurons and microglia (Supplementary Fig. 27).

**Fig. 6:**
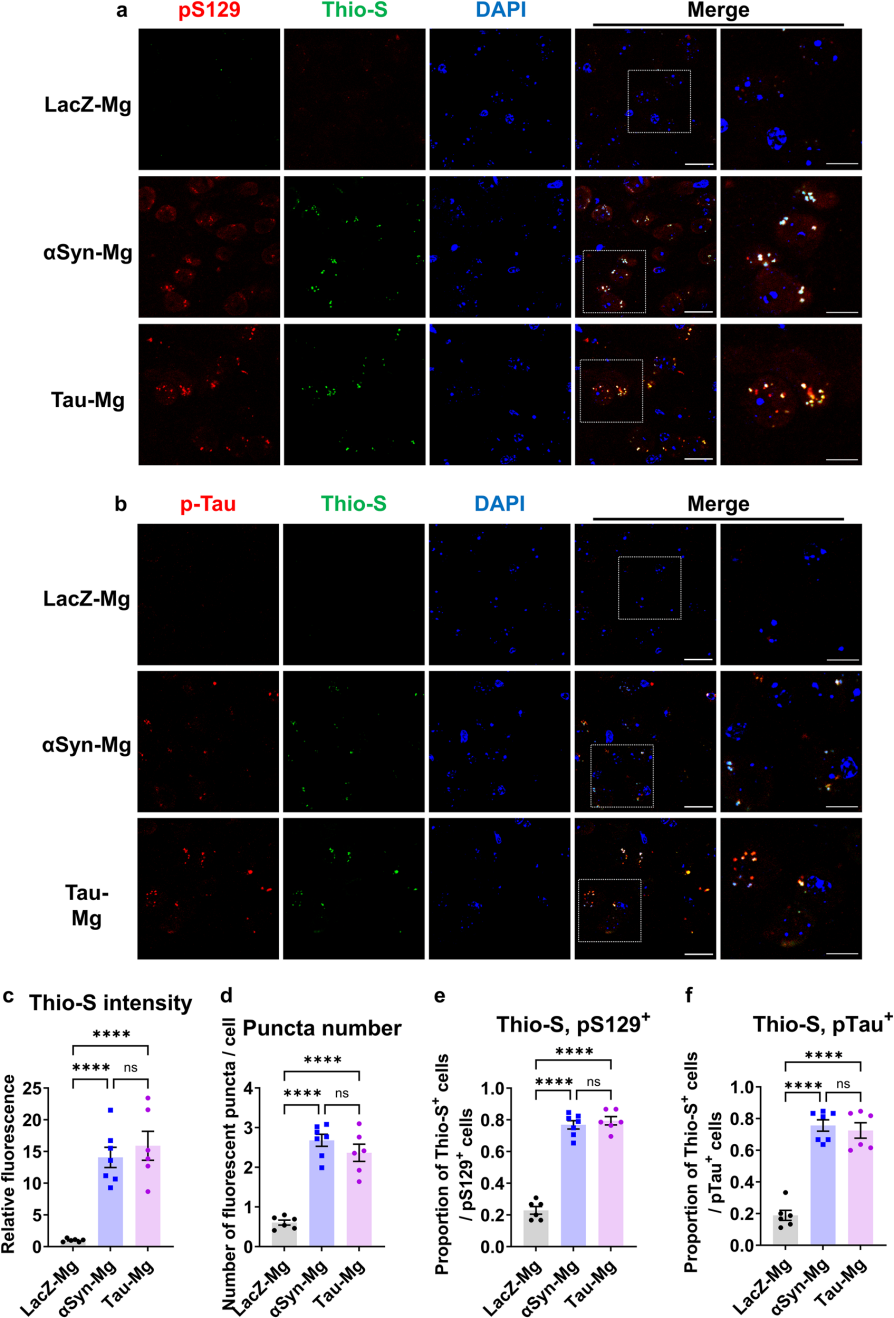
pS129 and p-tau inclusions are Thio-S positive. **a**, **b** Representative IF images of the ipsilateral motor cortex costained with Thio-S/pS129 (**a**) and Thio-S/p-tau (**b**) 1 month after injection. The ROI shown in the white box is magnified. Scale bars, 20 μm and 10 μm (for magnified images). **c**–**f** The relative intensity of Thio-S fluorescence (**c**), the number of fluorescent puncta per cell (**d**), the proportion of pS129^+^ cells immunoreactive with Thio-S (**e**) and the proportion of p-tau^+^ cells immunoreactive with Thio-S (**f**). All data are presented as the mean ± s.e.m. For statistical analysis, one-way ANOVA with Tukey’s post hoc test was performed.

To further characterize the structural properties of these protein aggregates, we conducted costaining of pS129 and p-tau with Thio-S, a dye that binds to β-sheet-rich structures. In both the αSyn-Mg and tau-Mg groups, we observed a marked increase in the relative Thio-S intensity, the number of fluorescent puncta per cell and the proportion of cells double-positive for Thio-S/pS129 or Thio-S/p-tau (Fig. 6a–f).

### Induction of gliosis and neuroinflammation in mice injected with activated microglia

**Fig. 7:**
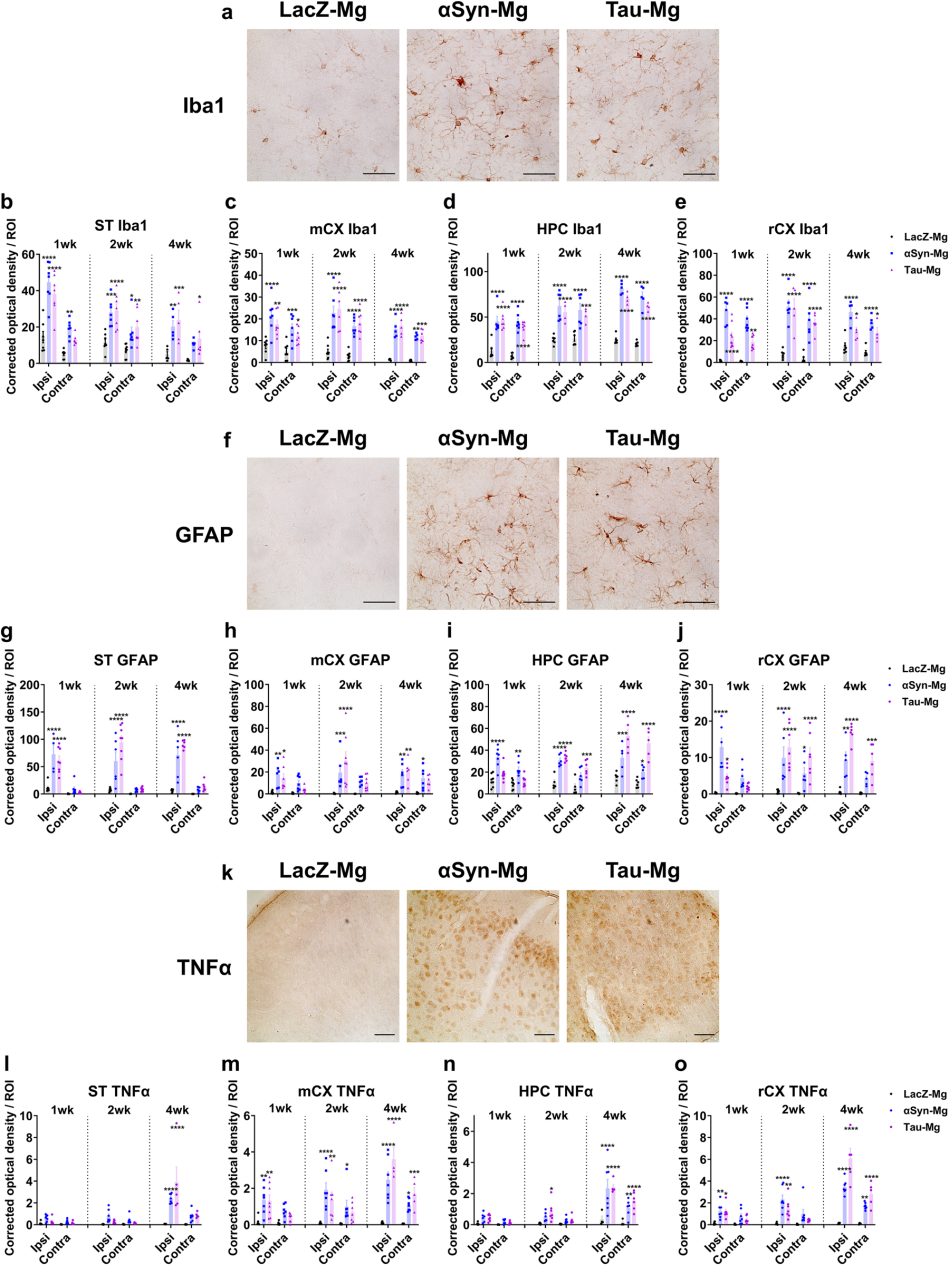
Gliosis and neuroinflammation are induced by transplantation of activated microglia. **a** Representative IHC images labeled with an antibody specific for Iba1 in the ipsilateral motor cortex 1 month after injection. Scale bar, 50 μm. **b**–**e** The relative optical density of Iba1 in the striatum (**b**), motor cortex (**c**), hippocampus (**d**) and rhinal cortex (**e**) 1, 2 and 4 weeks after injection. All data are presented as the mean ± s.e.m. For statistical analysis, two-way ANOVA followed by Tukey’s post hoc test was performed. **f** Representative IHC images labeled with an antibody specific for GFAP in the ipsilateral rhinal cortex 1 month after injection. Scale bar, 50 μm. **g**–**j** The relative optical density of GFAP in the striatum (**g**), motor cortex (**h**), hippocampus (**i**) and rhinal cortex (**j**) 1, 2 and 4 weeks after injection. All data are presented as the mean ± s.e.m. For statistical analysis, two-way ANOVA followed by Tukey’s post hoc test was performed. **k** Representative IHC images labeled with an antibody specific for TNFα in the ipsilateral rhinal cortex 1 month after injection. Scale bar, 100 μm. **l**–**o** The relative optical density of TNFα in the striatum (**l**), motor cortex (**m**), hippocampus (**n**) and rhinal cortex (**o**) 1, 2 and 4 weeks after injection. All data are presented as the mean ± s.e.m. For statistical analysis, two-way ANOVA followed by Tukey’s post hoc test was performed.

In addition to the emergence of compound proteinopathies in wide brain regions, we performed IHC with antibodies specific for Iba1, GFAP and TNFα to assess the extent of gliosis and neuroinflammation, which are key features in neurodegenerative diseases^39,40^. We observed notable increases in the OD values for Iba1 and GFAP in the αSyn-Mg and tau-Mg groups, indicating the induction of microgliosis and astrogliosis (Fig. 7a–j and Supplementary Figs. 28 and 29). The OD values for Iba1 in the ipsilateral striatum peaked at 1 week after injection and slightly decreased thereafter, while in other regions they either increased or remained stable over time (Fig. 7b–e). Regarding GFAP, the OD values significantly increased at 1 week after injection and remained at similar levels until 4 weeks after injection (Fig. 7g–j). The relative TNFα levels moderately but significantly increased at 1 and 2 weeks after injection, reaching their peak at 4 weeks after injection (Fig. 7k–o and Supplementary Fig. 30). As with the pS129 and p-tau patterns, the αSyn-Mg and tau-Mg groups demonstrated comparable distribution patterns of Iba1, GFAP and TNFα (Supplementary Figs. 28–30).

### Defects in motor and cognitive functions of mice injected with activated microglia

**Fig. 8:**
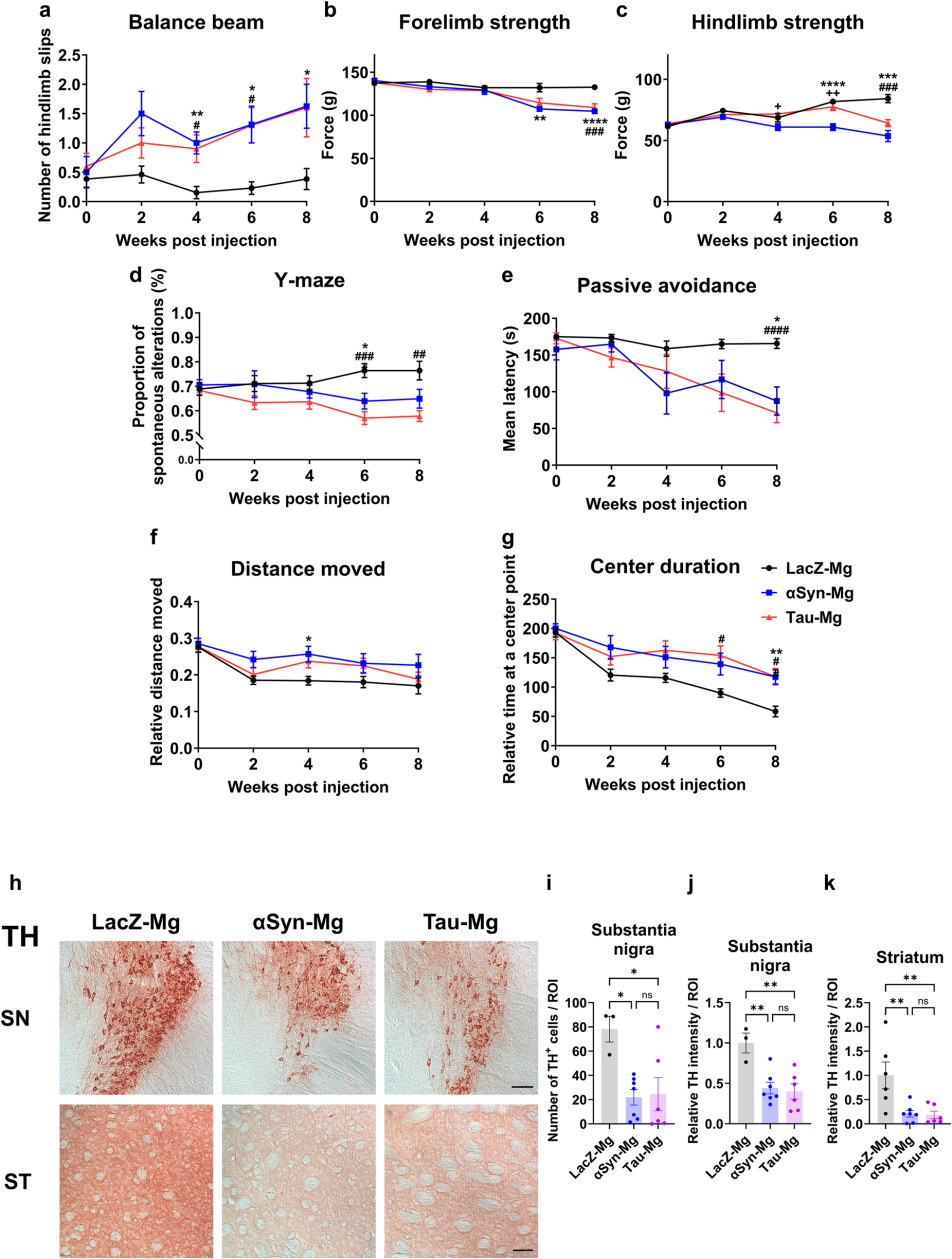
Motor and cognitive defects are shown in the mice injected with activated microglia. **a**–**c**, The number of hindlimb slips on a beam (**a**), and forelimb (**b**) and hindlimb (**c**) strength measured to evaluate motor functions of mice. **d**, **e** The proportion of spontaneous alternations in a Y-shaped apparatus (**d**) and the latency to enter a dark compartment where mice had experienced a foot shock (**e**) measured to evaluate cognitive functions of mice. **f**, **g** The relative distance moved (**f**) and relative time spent at a center point (**g**) in an open field. All data are presented as the mean ± s.e.m. For statistical analysis, two-way repeated measures ANOVA followed by Šidák’s post hoc test was performed. *LacZ-Mg versus αSyn-Mg; ^#^LacZ-Mg versus tau-Mg; ^+^αSyn-Mg versus tau-Mg. **h**–**k**, Representative IHC images of the SN and striatum (ST) labeled with an antibody specific for TH (**h**), quantifications of TH-positive cells in the SN (**i**) and relative TH levels in the SN (**j**) and ST (**k**). For **j** and **k**, values were normalized to the LacZ-Mg group. All data are presented as the mean ± s.e.m. For statistical analysis, one-way ANOVA with Tukey’s post hoc test was performed. Scale bar, 50 μm.

Lastly, we conducted a series of behavioral tests to assess the impact of compound proteinopathies, gliosis and neuroinflammation mediated by activated microglia on the motor and cognitive functions of mice. In both the αSyn-Mg and tau-Mg groups, there was a decrease in balance beam performance as early as 4 weeks after injection (Fig. 8a). Deficits in forelimb and hindlimb strength were also observed in the αSyn-Mg and tau-Mg groups at 6 and 8 weeks after injection, respectively (Fig. 8b,c). The hindlimb strength of mice in the αSyn-Mg group weakened more rapidly than in the tau-Mg group, and the differences between the two groups were statistically significant at weeks 6 and 8 (Fig. 8c). In the open field test, the moving distance of mice in the αSyn-Mg group significantly increased at 4 weeks after injection, and the time spent at the center point increased at 8 weeks after injection in both the αSyn-Mg and tau-Mg groups (Fig. 8f,g). There was no difference in body weight among the three groups (Supplementary Fig. 31). Given the significant changes observed in motor function, we investigated whether pathological alterations occurred in TH-positive dopaminergic neurons. We found a substantial reduction in the number of TH-positive cells in the SN, along with pronounced decreases in TH immunoreactivities in both the SN and striatum (Fig. 8h–k).

When assessing cognitive abilities, we observed defects in Y-maze performance in the αSyn-Mg and tau-Mg groups 6 weeks after injection (Fig. 8d). In the passive avoidance test, the latency to enter a dark compartment, where mice had previously experienced an electric shock, was reduced in the αSyn-Mg and tau-Mg groups 8 weeks after injection (Fig. 8e). Overall, our findings demonstrate that microglia activated by αSyn or tau cause widespread damage in the mouse brain, leading to the accumulation and propagation of pathogenic proteins, induction of gliosis and neuroinflammation, and motor and cognitive dysfunction.

## Discussion

In this study, we discovered that, when stimulated by αSyn or tau secreted from neuronal cells, microglia undergo a shift from a homeostatic to an inflammatory state and that transplanting these activated microglia into the striatum of naive mice resulted in the manifestation of neuropathological and behavioral phenotypes characteristic of neurodegenerative diseases. These phenotypes encompass widespread tauopathies and synucleinopathies, gliosis, neuroinflammation and cognitive and motor deficits. Importantly, the neuropathological features were not confined to the injection site but rather progressively extended to multiple brain regions. These findings indicate that microglia-driven neuroinflammation plays a crucial role in the onset and progression of neurodegenerative diseases during their early stages.

Mixed proteinopathies are commonly seen in neurodegenerative diseases. For instance, αSyn-positive LBs have been found in the amygdala of over half of patients with sporadic AD^41–43^. In addition, increased tau phosphorylation has been observed in the SN, striatum and frontal cortex of patients with PD^44–46^. In cases of dementia with LBs, AD-type pathologies such as amyloid plaques and neurofibrillary tangles are not uncommon^47–50^.

Cross-seeding between pathogenic proteins has been the main theory explaining the formation of mixed pathologies^51,52^. Our present study, along with our recent study where the injection of Aβ-activated microglia developed tauopathies and synucleinopathies in mice^53^, provides an alternative theory for the mixed pathology. That is, neuroinflammation triggered by microglia activation causes aggregation of multiple proteins in neurons. Previous studies suggested that this might be the case in humans as well. LBs developed in transplanted mesencephalic neurons in patients with PD, with microglial activation preceding the formation of LBs in these grafted tissues^54^. In patients with early-onset AD, inflammation tracers were colocalized with tau in the cortex and amygdala^55^. Importantly, neuroinflammation mediated by microglial activation was highly associated with dementia in both AD and PD cases^56,57^. In mice, it has been shown that neuroinflammation preceded the aggregation and spreading of αSyn, and that an anti-inflammatory treatment prevented synucleinopathies^22^. However, while our present study adds to the growing body of literature suggesting a causative role of neuroinflammation in the onset and progression of proteinopathies, further studies are still required to clarify the precise causality between neuroinflammation and protein aggregation.

scRNA-seq showed that microglia subclusters underwent changes when treated with αSyn-CM or tau-CM. In both groups, the population of homeostatic microglia decreased significantly, while the proportions of inflammatory microglia 1 and 2 increased substantially. These findings suggest that activated microglia adopted inflammatory traits. Both groups exhibited compound proteinopathies, as well as severe gliosis and neuroinflammation, when injected into the striatum of naive mice. This suggests that microglial activation alone is a strong driver of neuropathology, regardless of the stimulant. This finding is consistent with previous research showing that LPS-activated microglia produced nearly identical phenotypes^53^. Although both αSyn-CM-treated and tau-CM-treated microglia caused synucleinopathies and tauopathies, our results do not necessarily imply that all the protein aggregates elicit identical inflammatory responses. For example, western blot analyses in this study revealed potential differences in the spatial patterns of these proteinopathies depending on the stimulating protein. Further studies should investigate whether different protein stimuli, including αSyn and tau in this study and Aβ in our recent report, can polarize microglia into specific states that induce distinct proteinopathies^53^. In addition, adjusting factors such as protein dosage and treatment duration may be necessary to fully manifest the subtle differences in microglial responses. Overall, the heterogeneity of microglial responses to different protein stimulations remains a crucial issue to be solved.

We observed a progressive spreading of the pathological changes without any signs of exogenous microglia migrating out of the injection areas. This suggests that microglia have non-cell-autonomous effects that not only trigger changes in the injection sites but also contribute to the progression of pathology in wider brain regions. There is a growing body of evidence supporting the role of inflammation in protein aggregation and the spread of aggregates. Tau aggregation has been shown to be induced by NLRP3 inflammasome^25,58^, while neuroinflammation promotes αSyn aggregation and spreading^22,24^. Conversely, protein aggregates can induce neuroinflammation through the activation of microglia. Neuron-released oligomeric αSyn activates microglia, causing pro-inflammatory responses^37^. Furthermore, tau induces the transformation of microglia into inflammatory states through the TLR2 signaling pathway^59^. Taking into consideration the interplay between neuroinflammation and protein aggregation, we propose the following model to explain how inflammation leads to protein aggregation and how the pathology (both protein aggregation and inflammation) spreads through the brain regions: Injection of activated microglia creates an inflammatory microenvironment near the injection site, where neurons form αSyn and tau aggregates. The protein aggregates are then secreted from neurons, further activating nearby microglia and exacerbating inflammation, thus establishing a vicious cycle between inflammation and protein aggregation. The protein aggregates within neurons then travel through the neural processes to distant brain regions, where they are secreted and establish additional vicious cycles between inflammation and protein aggregation.

In conclusion, we have demonstrated that injecting microglial cells that have been activated by αSyn or tau proteins is sufficient to induce all the characteristic features of neurodegenerative diseases, such as synucleinopathy and tauopathy, gliosis, neuroinflammation and both motor and cognitive deficits. As the disease progresses, the pathological features, including protein aggregation and glia activation, spread throughout different regions of the brain. We believe that the microglia injection model established in this study will serve as a novel and powerful tool for investigating the interactions between neuroinflammation and protein aggregation in neurodegenerative diseases. Gaining a better understanding of the mechanisms that link neuroinflammation to protein aggregation could reveal new targets for therapeutic interventions in treating neurodegenerative diseases.

## Supplementary information

Supplementary Information

Supplementary Table 1

Supplementary Table 2

## Data Availability

All data are available within the Article or its Supplementary Information.
