# Supplementary material for: Microglia-driven inflammation induces progressive tauopathies and synucleinopathies
Source: Exp Mol Med. 2025 May 1;57(5):1017–31. doi: 10.1038/s12276-025-01450-z (PMC12130470; doi:10.1038/s12276-025-01450-z)
Supplement: Supplementary file 1 — Supplementary Information [file 12276_2025_1450_MOESM1_ESM.pdf]

Supplementary Fig. 1

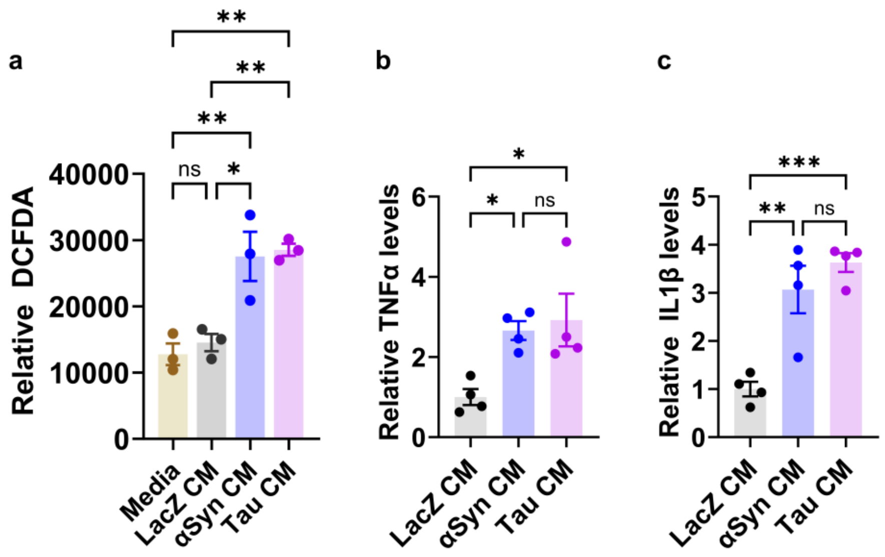

Supplementary Fig. 1: Induction of reactive oxygen species and proinflammatory cytokines in microglia exposed to α-synuclein and Tau. a The relative DCFDA fluorescence detecting intracellular reactive oxygen species levels. **b-c** The relative mRNA levels of *TNFα* (**b**) and *IL1β* (**c**).

Supplementary Fig. 2

a

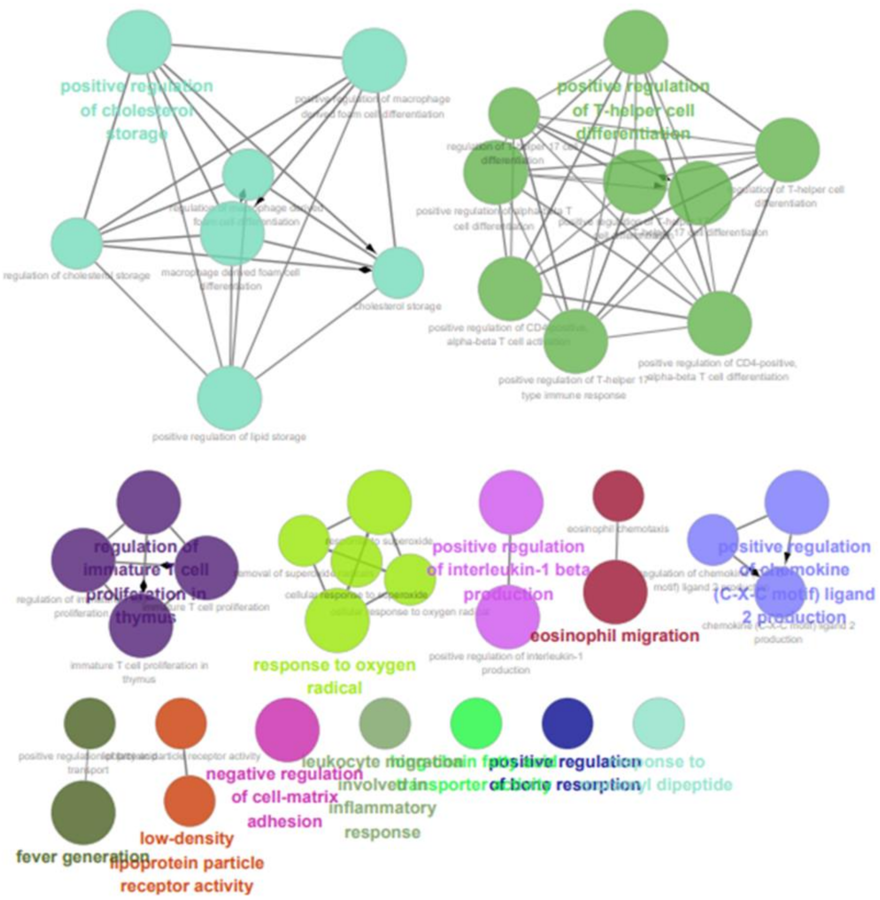

b

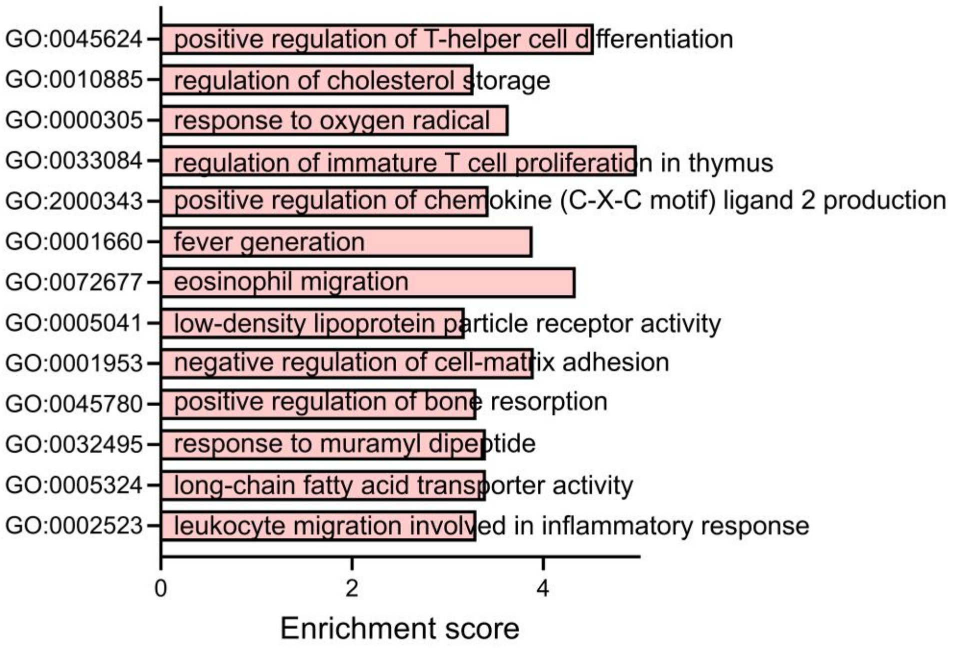

Supplementary Fig. 2: Molecular features of inflammatory microglia 1. a A simplified network of significantly enriched GO terms in inflammatory microglia 1 using 372 feature genes. Each term in the network is statistically significant (Benjamini-Hochberg correction < 0.05). **b** The top enriched GO terms in inflammatory microglia 1.

Supplementary Fig. 3

a

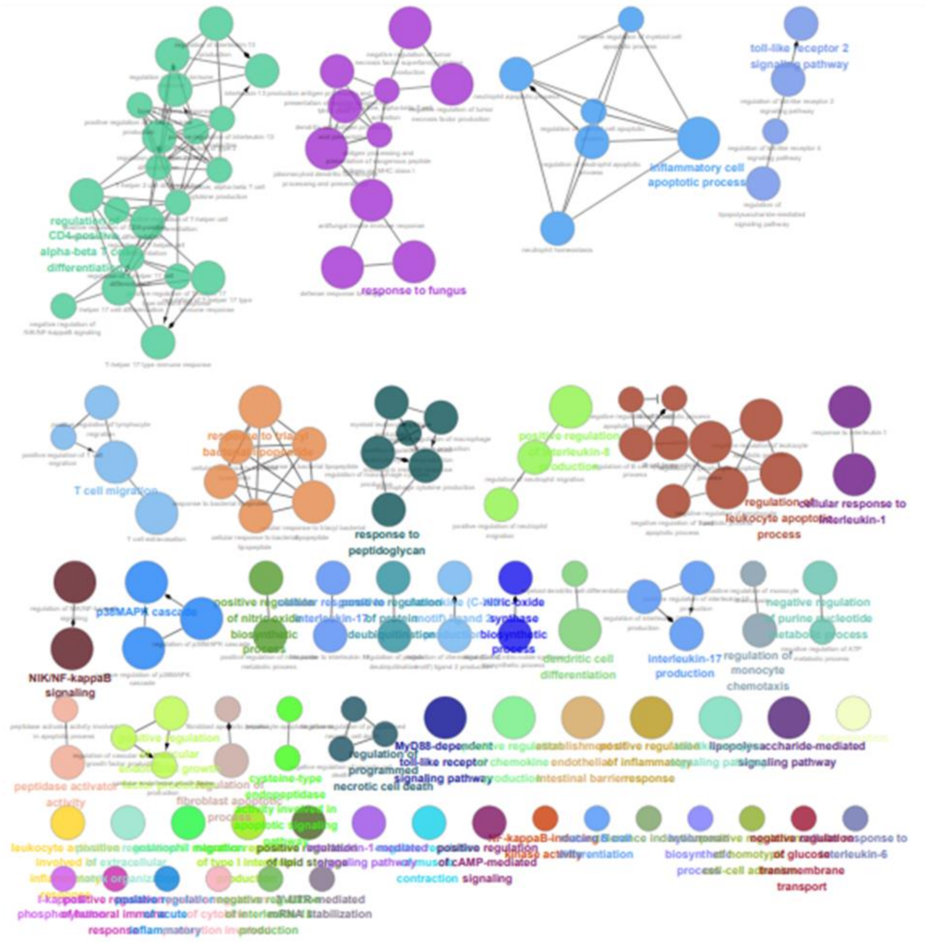

b

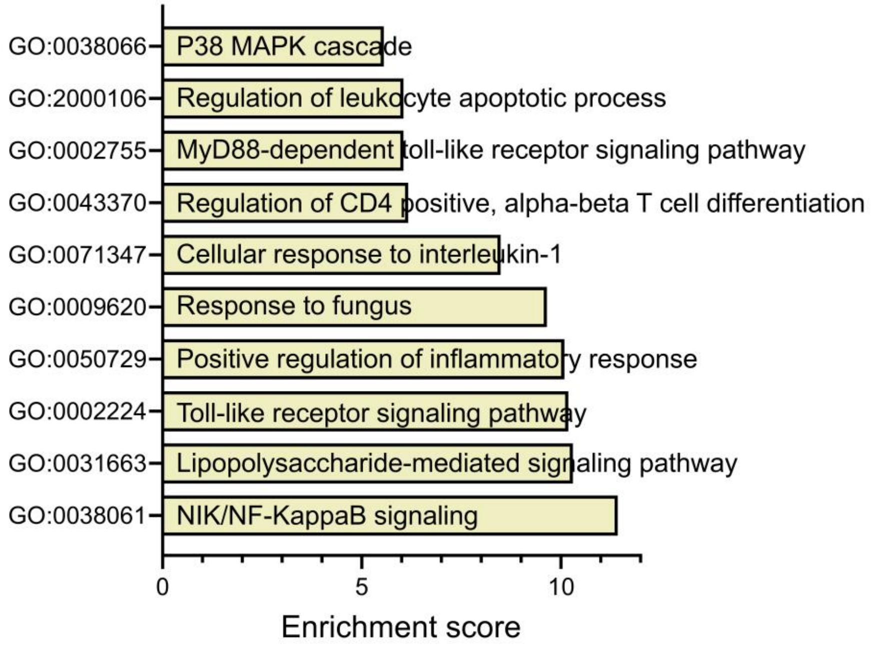

Supplementary Fig. 3: Molecular features of inflammatory microglia 2. a A simplified network of significantly enriched GO terms in inflammatory microglia 2 using 769 feature genes. Each term in the network is statistically significant (Benjamini-Hochberg correction < 0.05). **b** The top enriched GO terms in inflammatory microglia 2.

Supplementary Fig. 4 Expression levels of feature genes (Top 90)

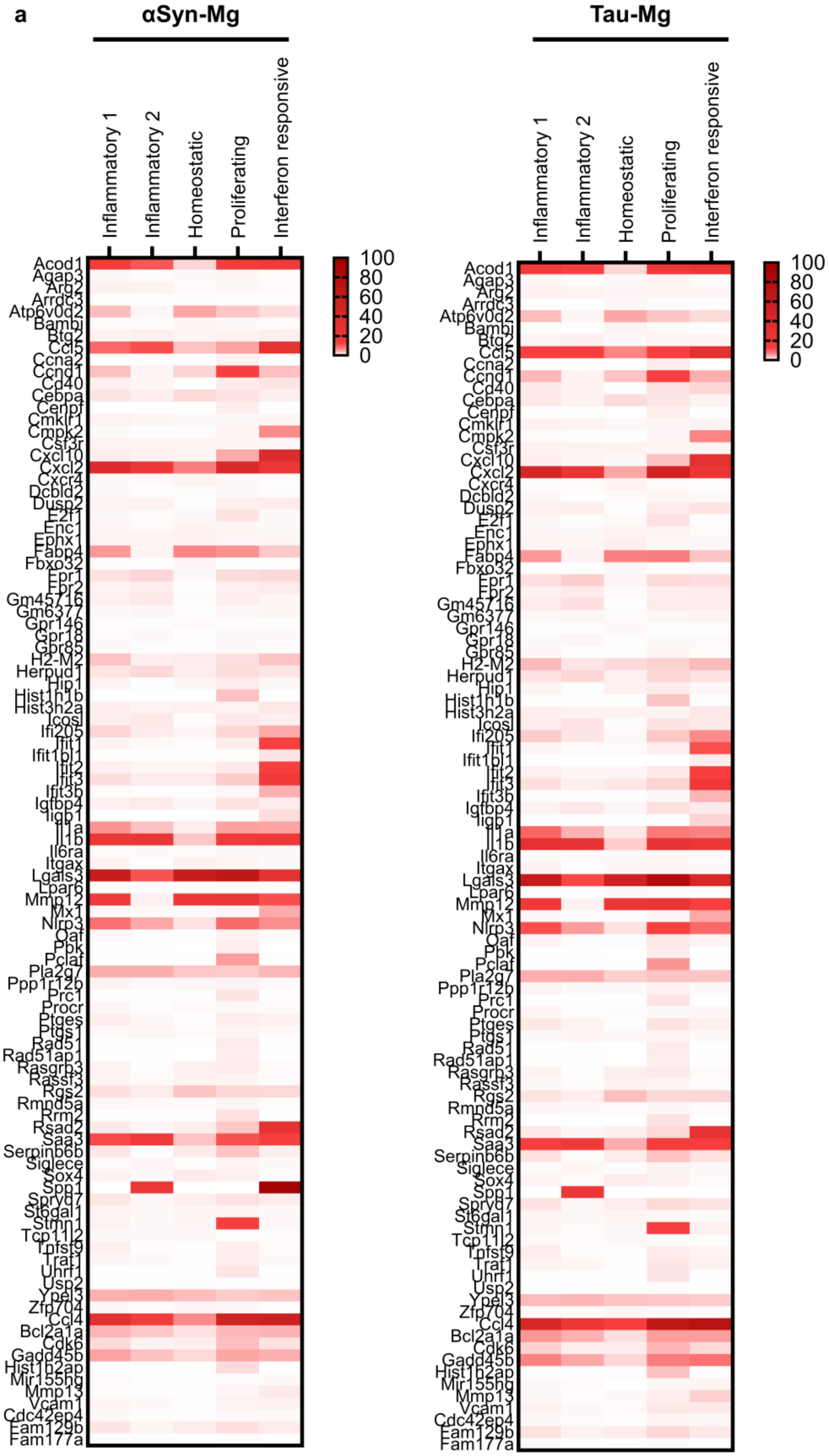

Supplementary Fig. 4: Expression levels for the top 90 feature genes. a A heatmap illustrating the expression of variable genes (FDR < 0.05) in microglia subclusters treated with αSyn or Tau.

Supplementary Fig. 5

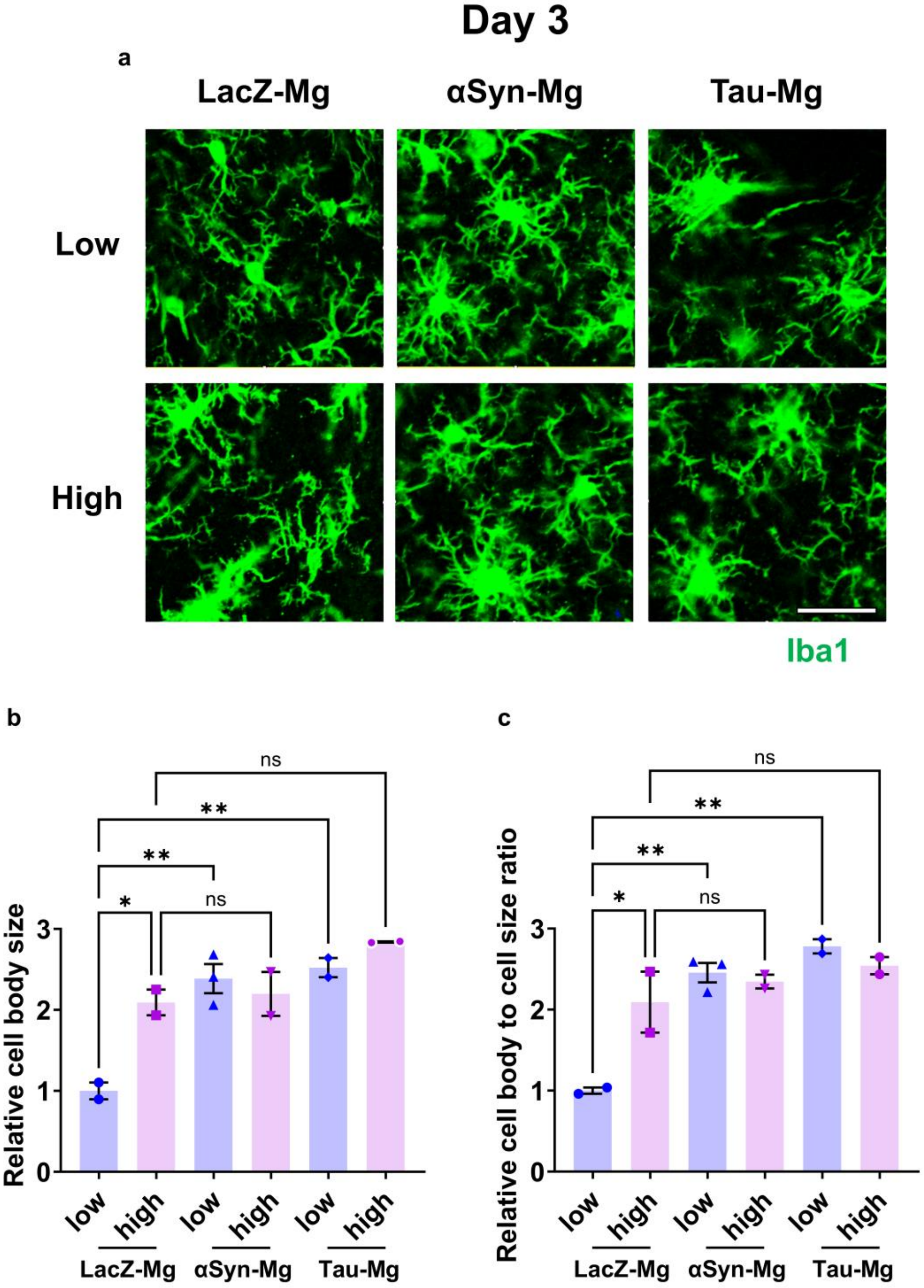

Supplementary Fig. 5: Determining the number of microglia to be injected into the striatum. a Representative IF images of the ipsilateral striatum labeled with an antibody specific for Iba1 3 days after injection. Low number (2 x 10^4 cells) or high number (5 x 10^4 cells) of microglia were injected. Scale bar, 30 μm. **b-c** Relative cell body size (**b**) and cell body to cell size ratio (**c**). All data are presented as the means ± SEMs. For statistical analysis, one-way ANOVA with Tukey's post hoc test was performed.

Supplementary Fig. 6

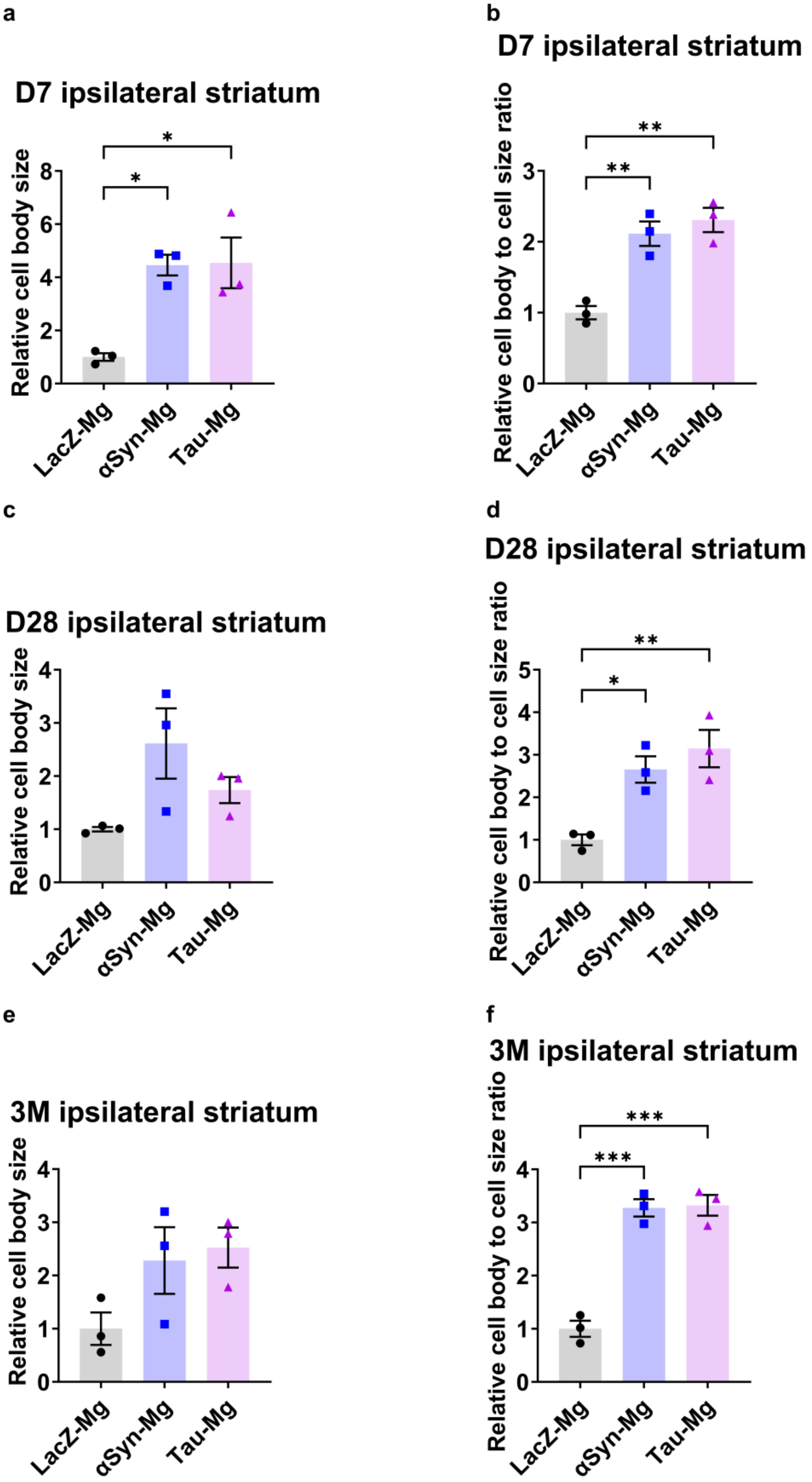

Supplementary Fig. 6: Activation of endogenous microglia after intrastriatal injection of activated microglia. a-f Relative cell body size and cell body to cell size ratio 7 days (**a-b**), 28 days (**c-d**), and 3 months (**e-f**) after injection using Iba1 immunofluorescence. 2 x 10^4 microglial cells were injected. All data are presented as the means ± SEMs. For statistical analysis, one-way ANOVA with Tukey's post hoc test was performed.

Supplementary Fig. 7

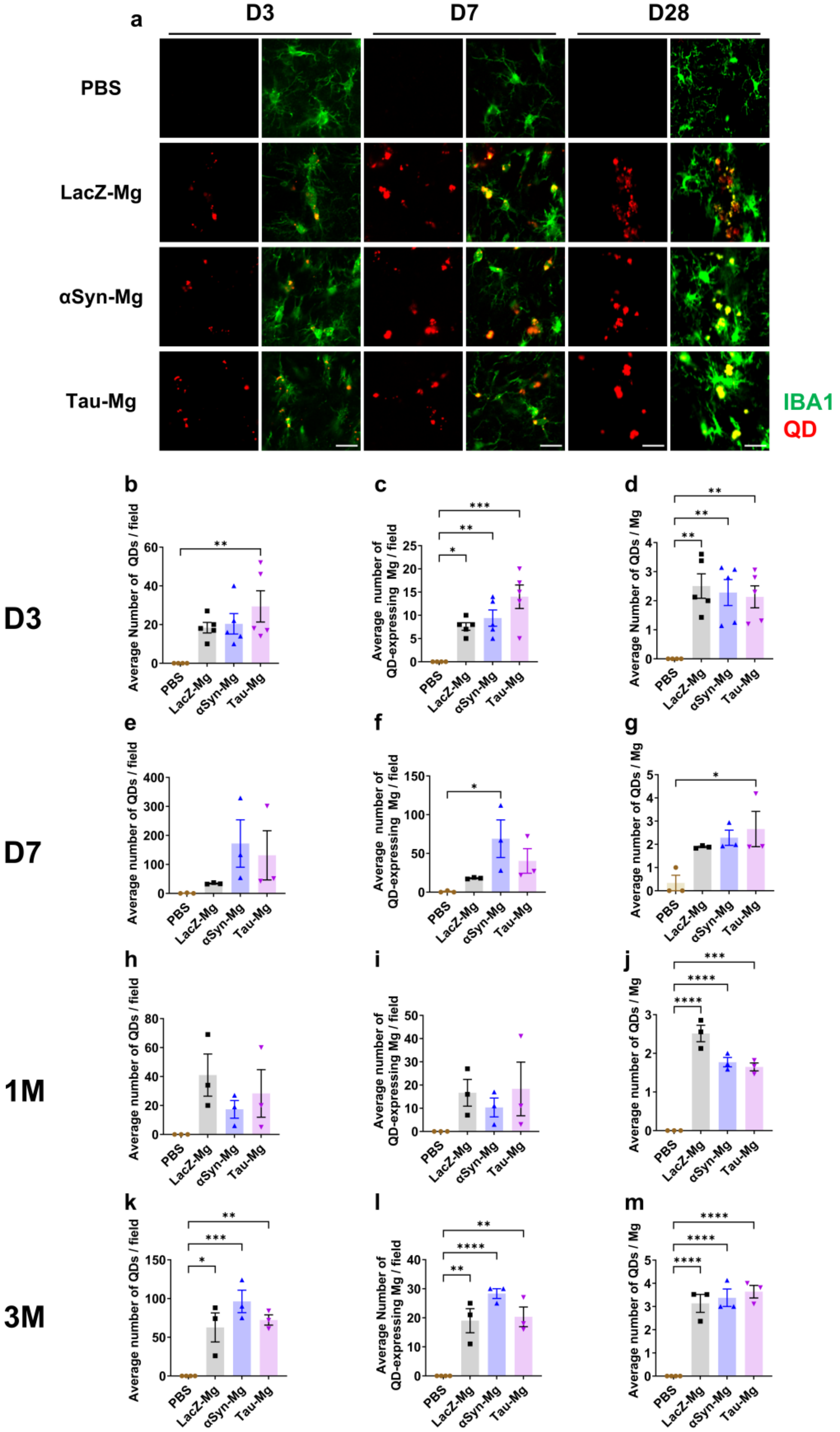

Supplementary Fig. 7: Distribution of activated microglia labeled with quantum dots. a Representative IF images of the ipsilateral striatum labeled with an antibody specific for Iba1 3 days, 7 days, and 28 days after injection of activated microglia pretreated with QDs. Scale bar, 30 μm. **b-m** The number of QDs, QD-expressing microglia, and QDs per microglia counted 3 days (**b-d**), 7 days (**e-g**), 1 month (**h-j**), and 3 months (**k-m**) after injection. All data are presented as the means ± SEMs. For statistical analysis, one-way ANOVA with Tukey's post hoc test was performed.

Supplementary Fig. 8

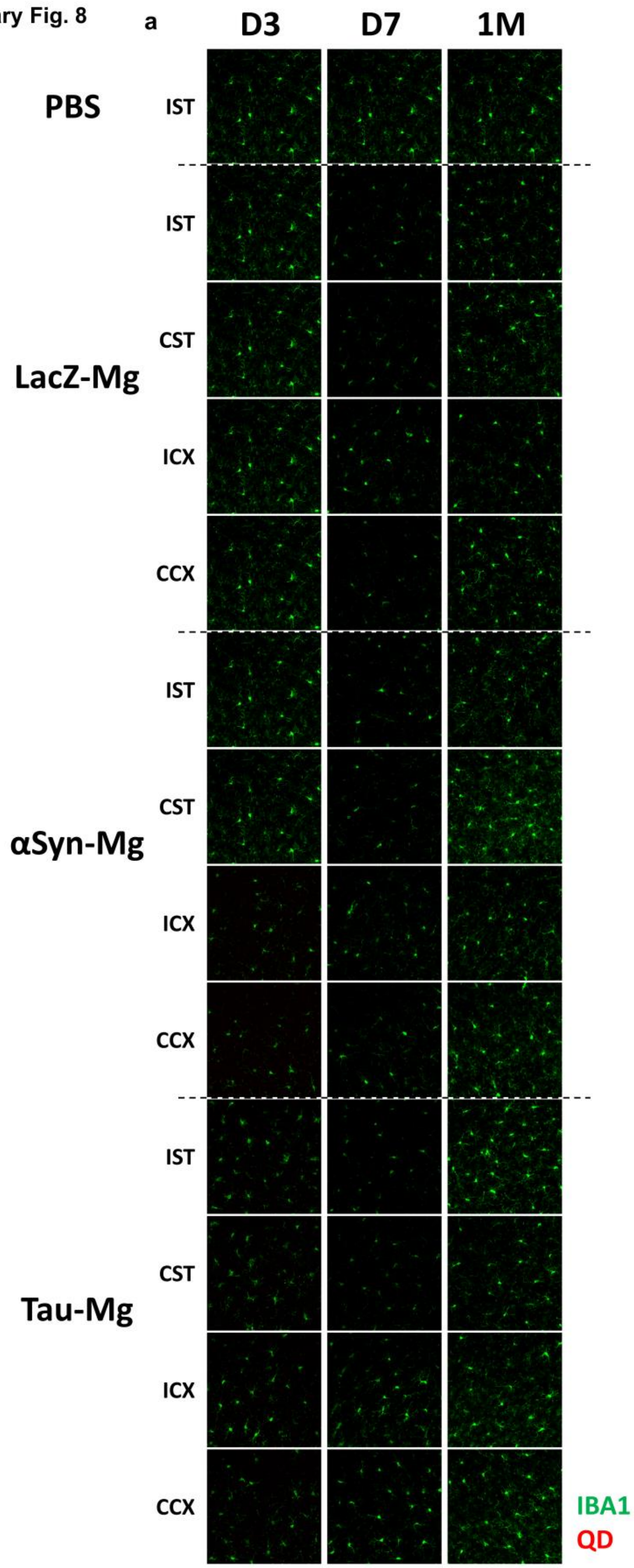

Supplementary Fig. 8: Detection of quantum dot signals in the brain regions distal to injection areas. a Representative IF images of the brain regions labeled with an antibody specific for Iba1 3 days, 7 days, and 1 month after injection of activated microglia pretreated with QDs. Scale bar, 50 μm. IST, ipsilateral striatum distal to injection area; CST, contralateral striatum; ICX, ipsilateral motor cortex; CCX, contralateral motor cortex.

Supplementary Fig. 9

a

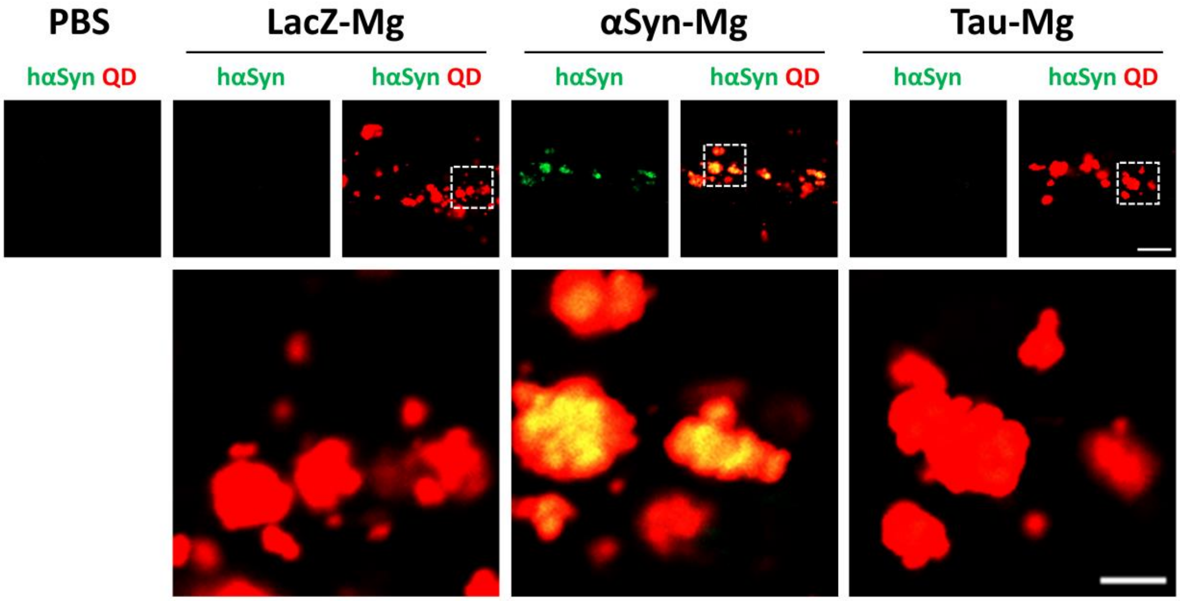

b

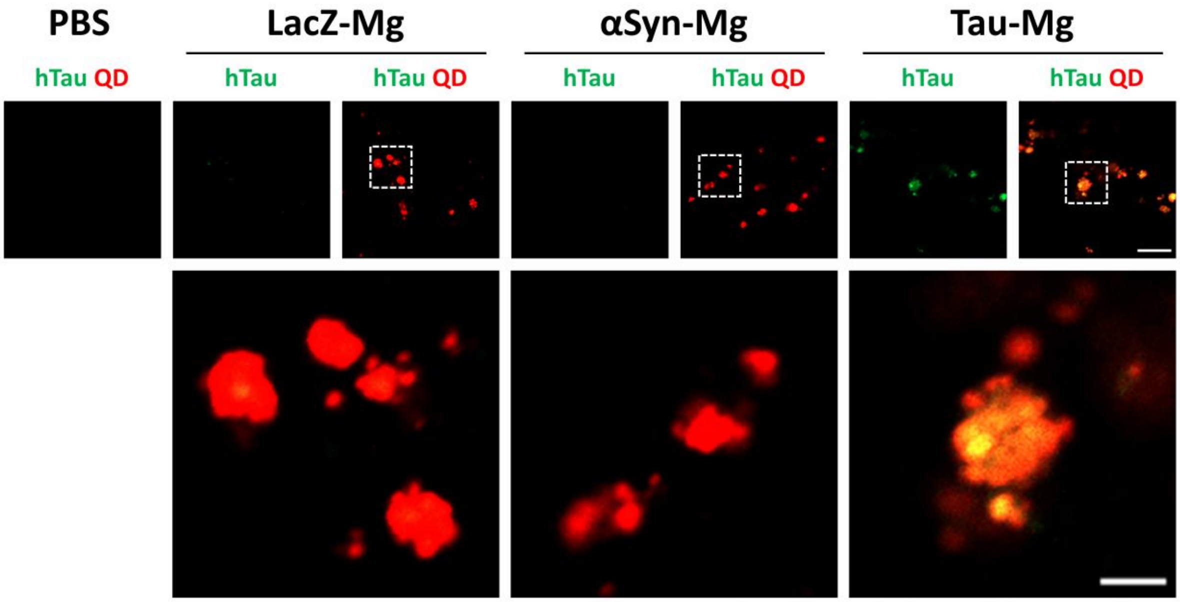

Supplementary Fig. 9: Costaining of quantum dots with human forms of αSyn or Tau in the ipsilateral striatum. a Representative IF images of the ipsilateral striatum labeled with an antibody specific for human αSyn, 3 months after the injection of activated microglia pretreated with QDs. ROI shown in the white box is magnified. Scale bar, 20 μm; and 5 μm for magnified images. **b** Representative IF images of the ipsilateral striatum labeled with an antibody specific for human Tau, 3 months after the injection of activated microglia pretreated with QDs. ROI shown in the white box is magnified. Scale bar, 20 μm; and 5 μm for magnified images.

Supplementary Fig. 10

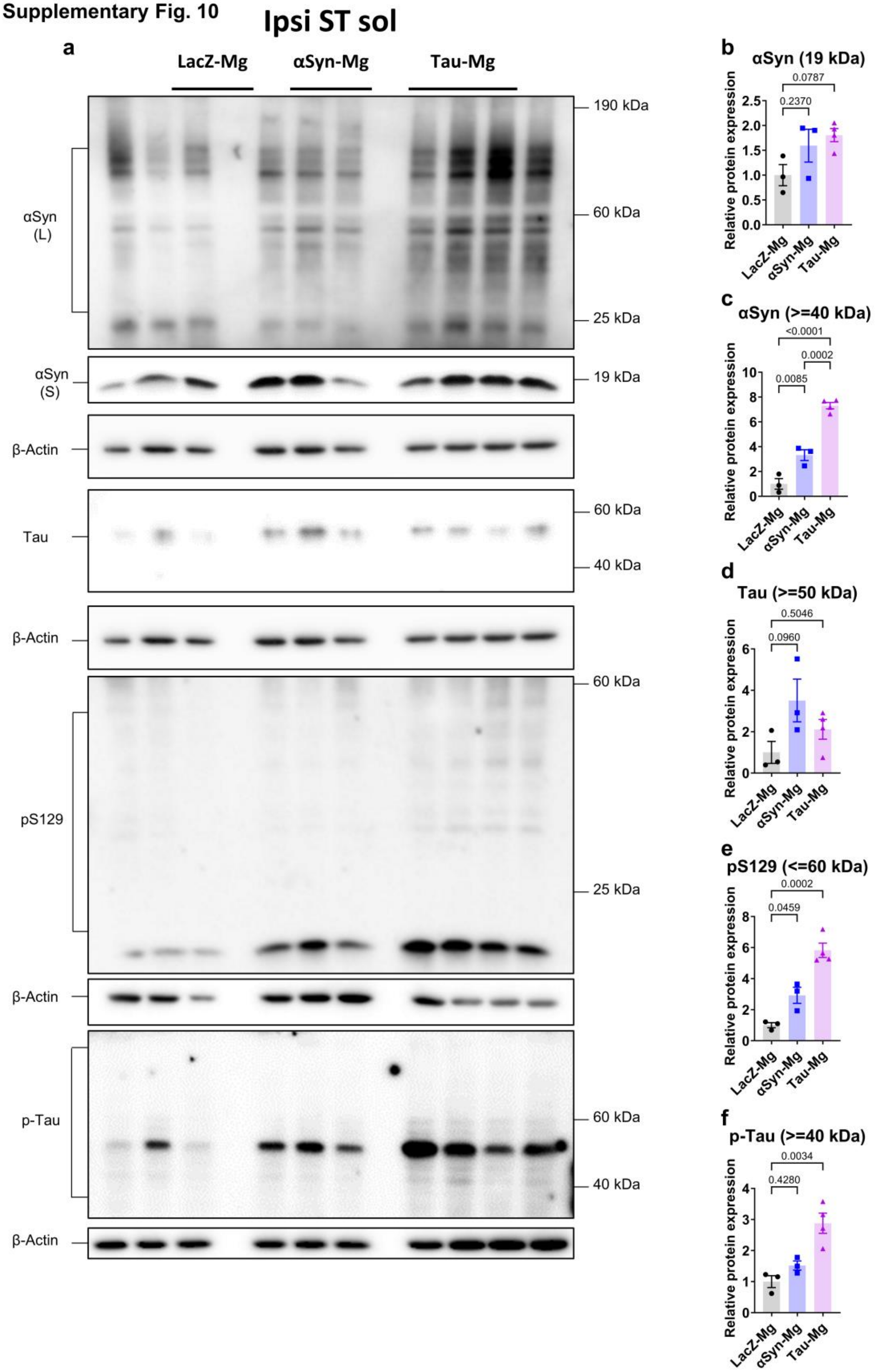

Supplementary Fig. 10: Quantification of the relative protein levels in the Tx-sol fraction of the ipsilateral striatum. a Representative western blot images of the Tx-sol fraction of the ipsilateral striatum 1 month after injection. L, long exposure time; S, short exposure time. **b-f** The relative expression levels of monomeric α-synuclein (**b**), oligomeric α-synuclein (**c**), Tau (**d**), pS129 (**e**), and p-Tau (**f**). All data are presented as the means ± SEMs. For statistical analysis, one-way ANOVA with Tukey's post hoc test was performed.

Supplementary Fig. 11

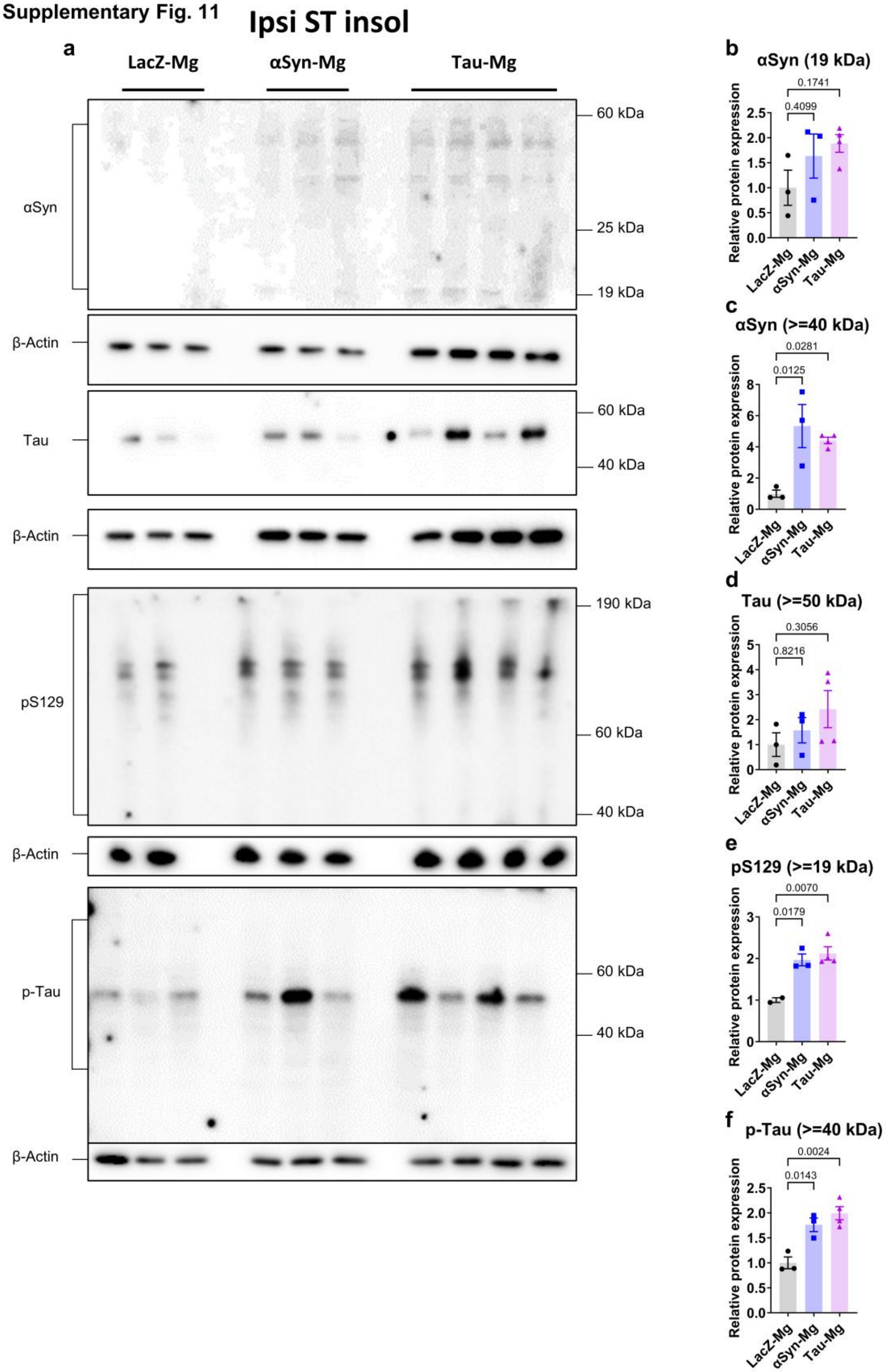

Supplementary Fig. 11: Quantification of the relative protein levels in the Tx-insol fraction of the ipsilateral striatum. a Representative western blot images of the Tx-insol fraction of the ipsilateral striatum 1 month after injection. L, long exposure time; S, short exposure time. **b-f** The relative expression levels of monomeric α-synuclein (**b**), oligomeric α-synuclein (**c**), Tau (**d**), pS129 (**e**), and p-Tau (**f**). All data are presented as the means ± SEMs. For statistical analysis, one-way ANOVA with Tukey's post hoc test was performed.

Supplementary Fig. 12

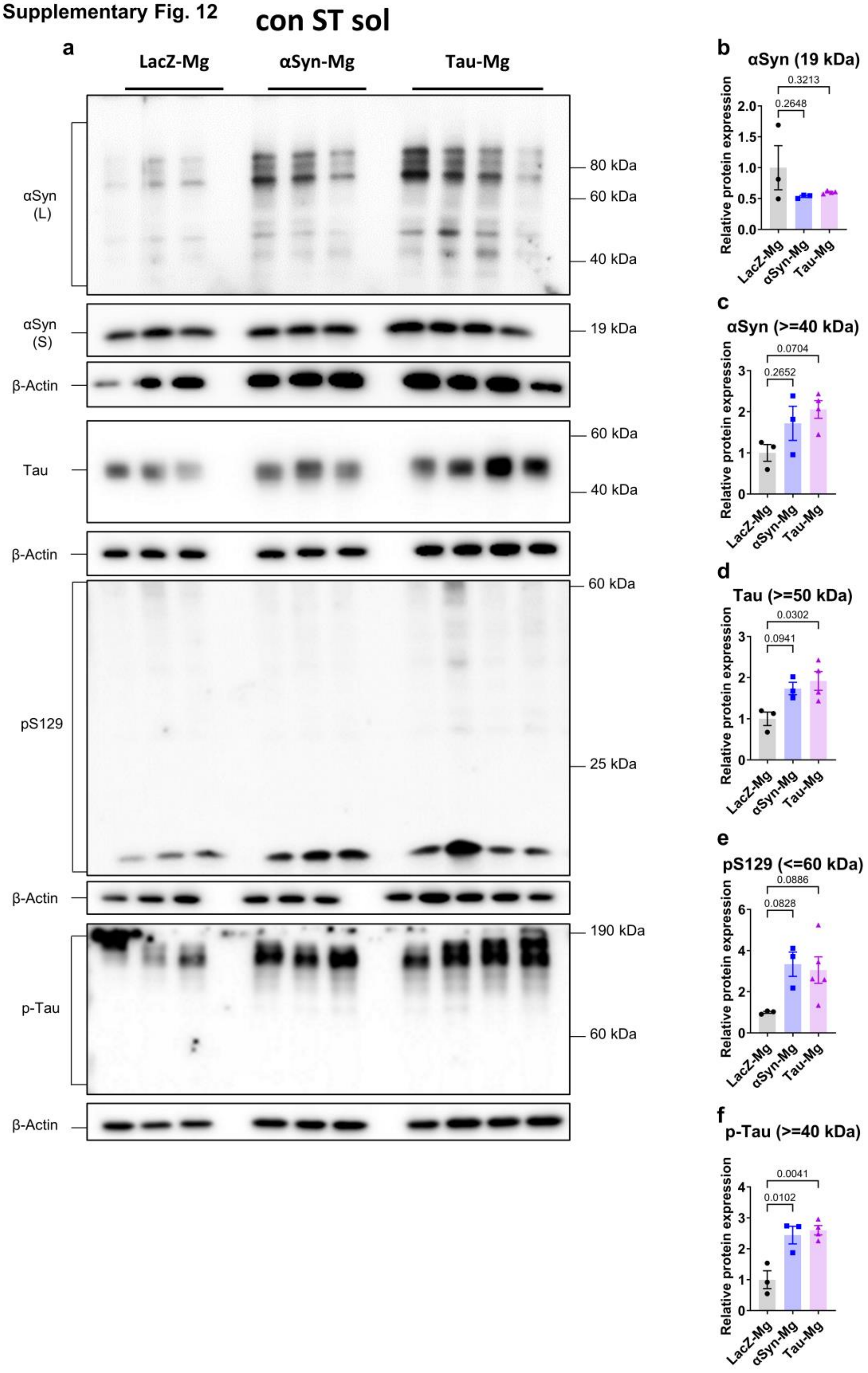

Supplementary Fig. 12: Quantification of the relative protein levels in the Tx-sol fraction of the contralateral striatum. a Representative western blot images of the Tx-sol fraction of the contralateral striatum 1 month after injection. L, long exposure time; S, short exposure time. **b-f** The relative expression levels of monomeric α-synuclein (**b**), oligomeric α-synuclein (**c**), Tau (**d**), pS129 (**e**), and p-Tau (**f**). All data are presented as the means ± SEMs. For statistical analysis, one-way ANOVA with Tukey's post hoc test was performed.

Supplementary Fig. 13

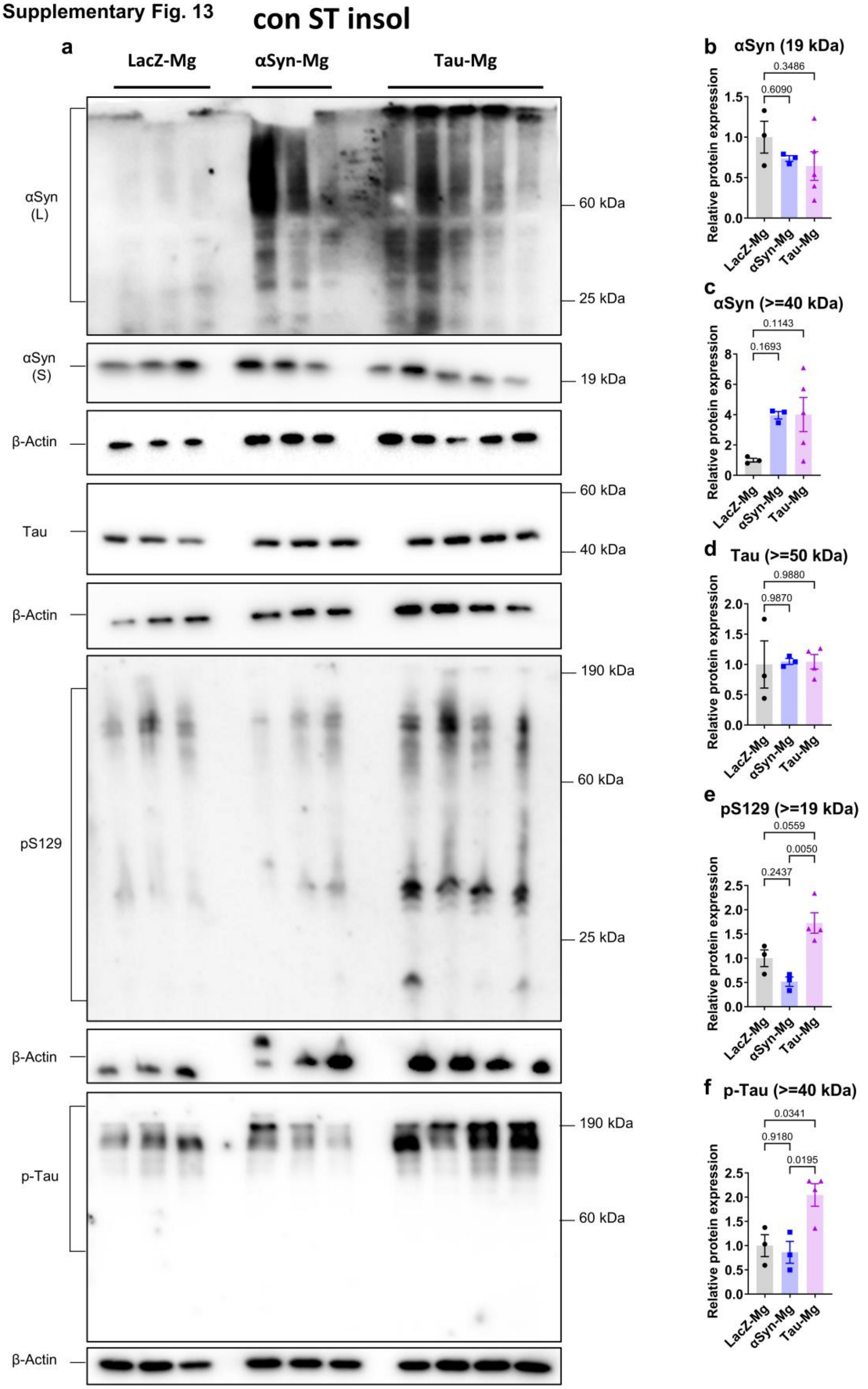

Supplementary Fig. 13: Quantification of the relative protein levels in the Tx-insol fraction of the contralateral striatum. a Representative western blot images of the Tx-insol fraction of the contralateral striatum 1 month after injection. L, long exposure time; S, short exposure time. **b-f** The relative expression levels of monomeric α-synuclein (**b**), oligomeric α-synuclein (**c**), Tau (**d**), pS129 (**e**), and p-Tau (**f**). All data are presented as the means ± SEMs. For statistical analysis, one-way ANOVA with Tukey's post hoc test was performed.

Supplementary Fig. 14

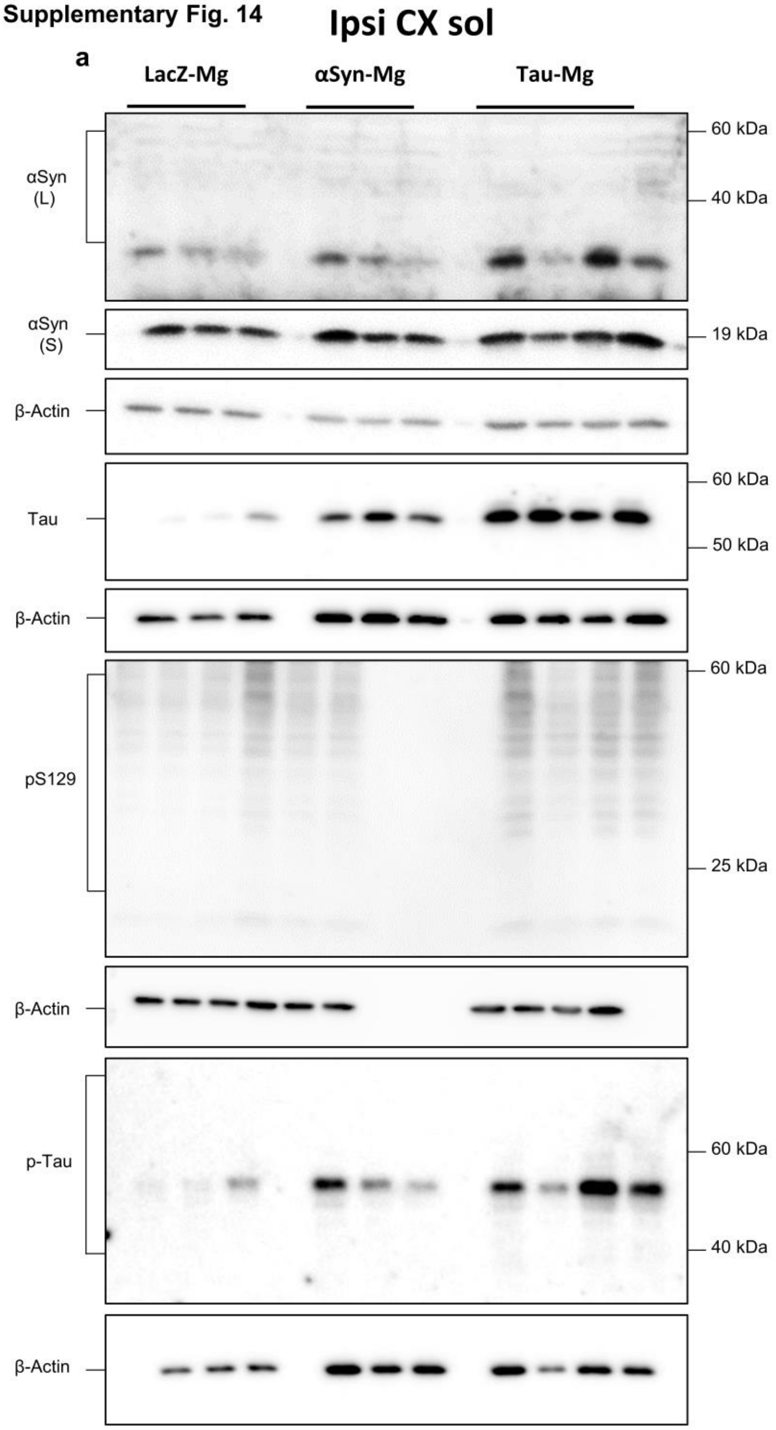

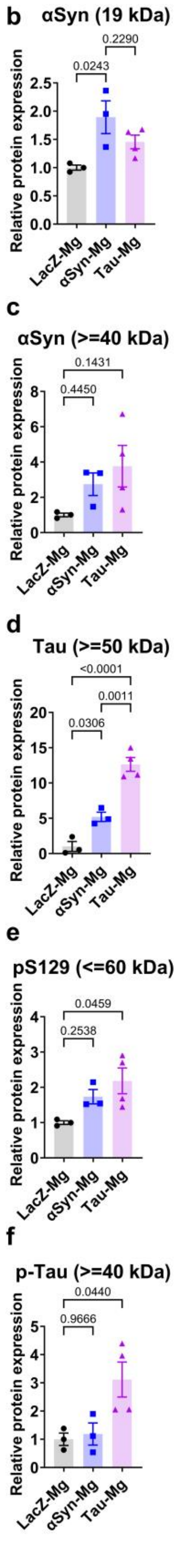

Supplementary Fig. 14: Quantification of the relative protein levels in the Tx-sol fraction of the ipsilateral cortex. a Representative western blot images of the Tx-sol fraction of the ipsilateral cortex 1 month after injection. L, long exposure time; S, short exposure time. **b-f** The relative expression levels of monomeric α-synuclein (**b**), oligomeric α-synuclein (**c**), Tau (**d**), pS129 (**e**), and p-Tau (**f**). All data are presented as the means ± SEMs. For statistical analysis, one-way ANOVA with Tukey's post hoc test was performed.

Supplementary Fig. 15

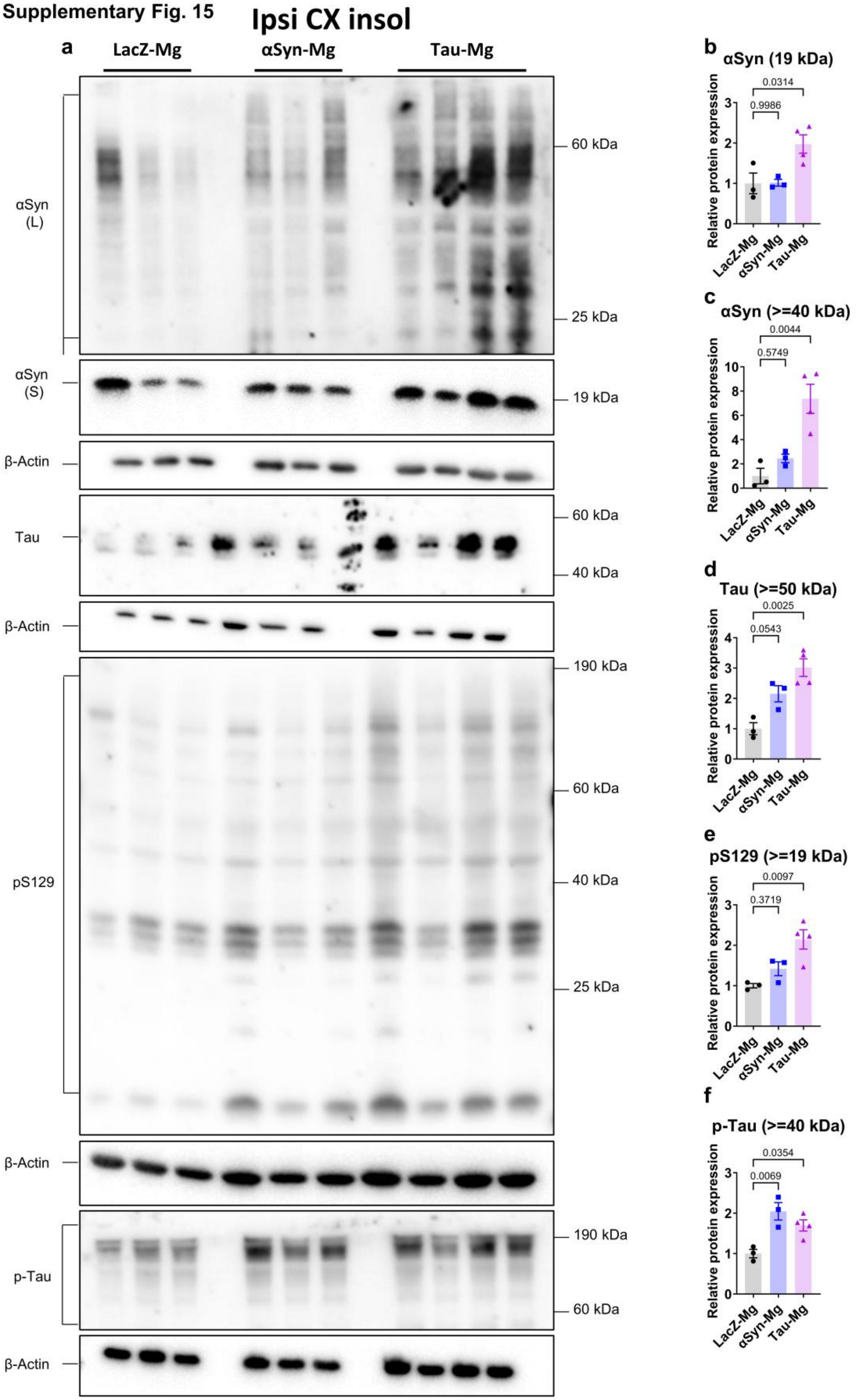

Supplementary Fig. 15: Quantification of the relative protein levels in the Tx-insol fraction of the ipsilateral cortex. a Representative western blot images of the Tx-insol fraction of the ipsilateral cortex 1 month after injection. L, long exposure time; S, short exposure time. **b-f** The relative expression levels of monomeric α-synuclein (**b**), oligomeric α-synuclein (**c**), Tau (**d**), pS129 (**e**), and p-Tau (**f**). All data are presented as the means ± SEMs. For statistical analysis, one-way ANOVA with Tukey's post hoc test was performed.

Supplementary Fig. 16

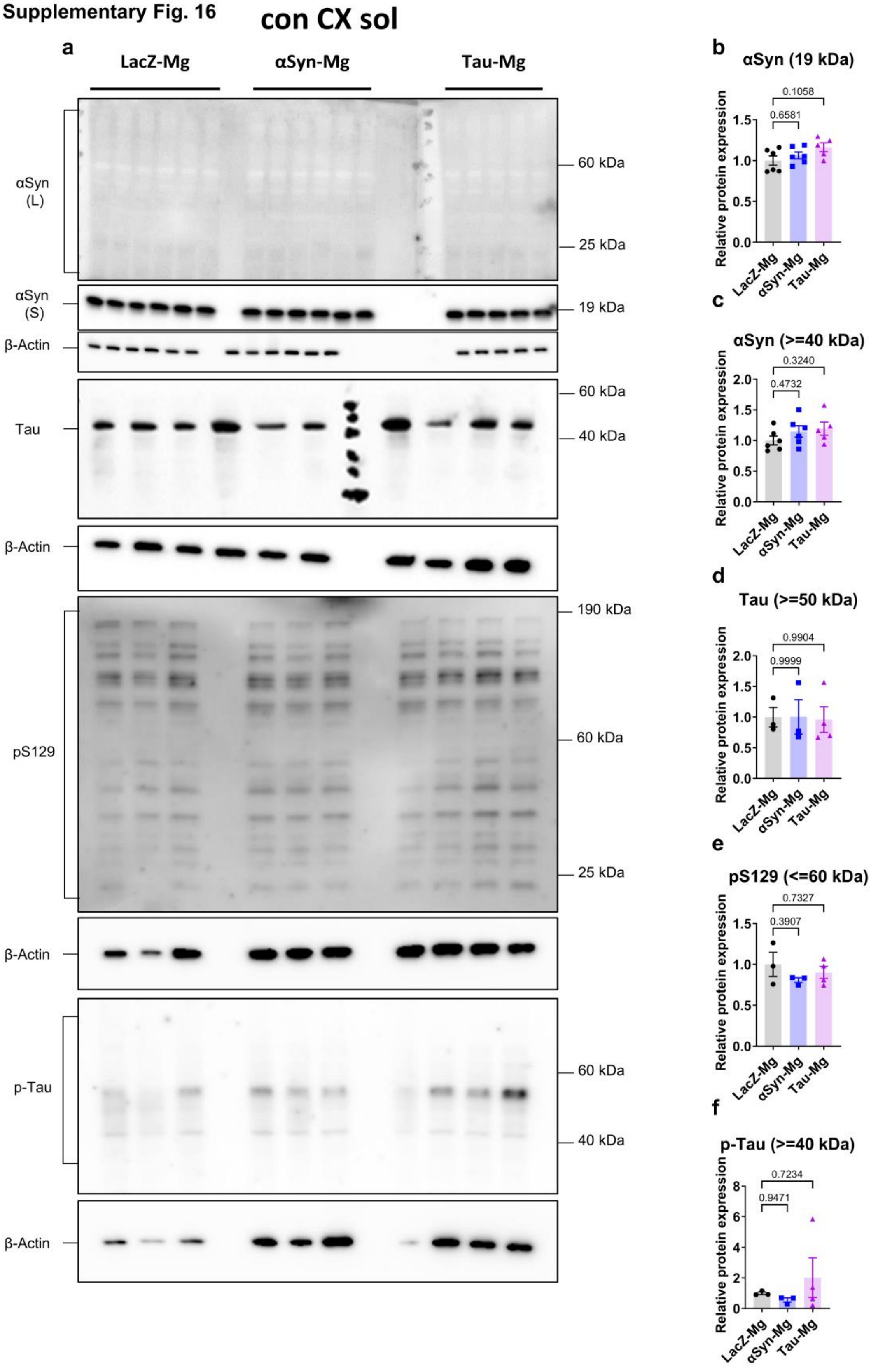

Supplementary Fig. 16: Quantification of the relative protein levels in the Tx-sol fraction of the contralateral cortex. a Representative western blot images of the Tx-sol fraction of the contralateral cortex 1 month after injection. L, long exposure time; S, short exposure time. **b-f** The relative expression levels of monomeric α-synuclein (**b**), oligomeric α-synuclein (**c**), Tau (**d**), pS129 (**e**), and p-Tau (**f**). All data are presented as the means ± SEMs. For statistical analysis, one-way ANOVA with Tukey's post hoc test was performed.

Supplementary Fig. 17

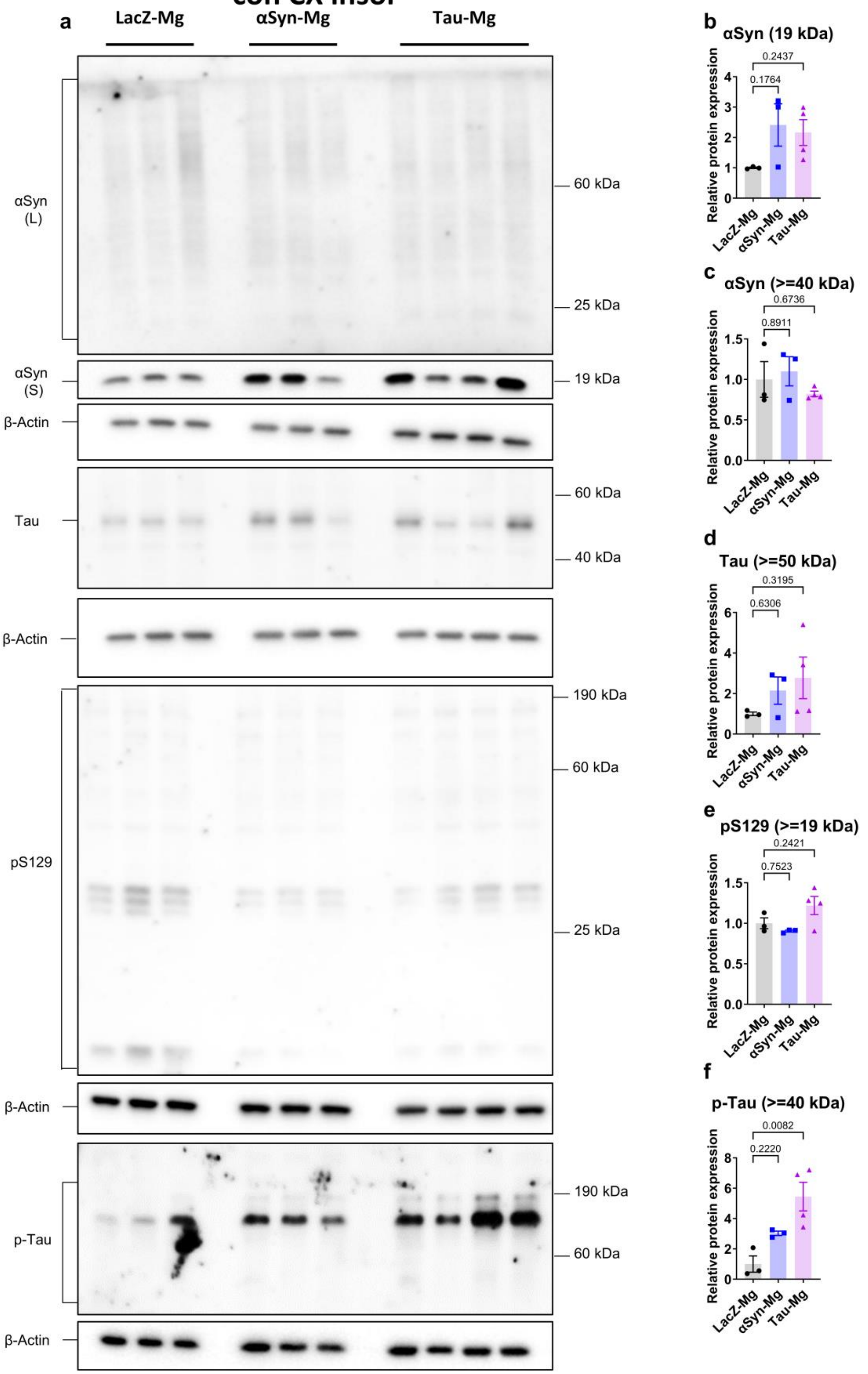

Supplementary Fig. 17: Quantification of the relative protein levels in the Tx-insol fraction of the contralateral cortex. a Representative western blot images of the Tx-insol fraction of the contralateral cortex 1 month after injection. L, long exposure time; S, short exposure time. **b-f** The relative expression levels of monomeric α-synuclein (**b**), oligomeric α-synuclein (**c**), Tau (**d**), pS129 (**e**), and p-Tau (**f**). All data are presented as the means ± SEMs. For statistical analysis, one-way ANOVA with Tukey's post hoc test was performed.

Supplementary Fig. 18

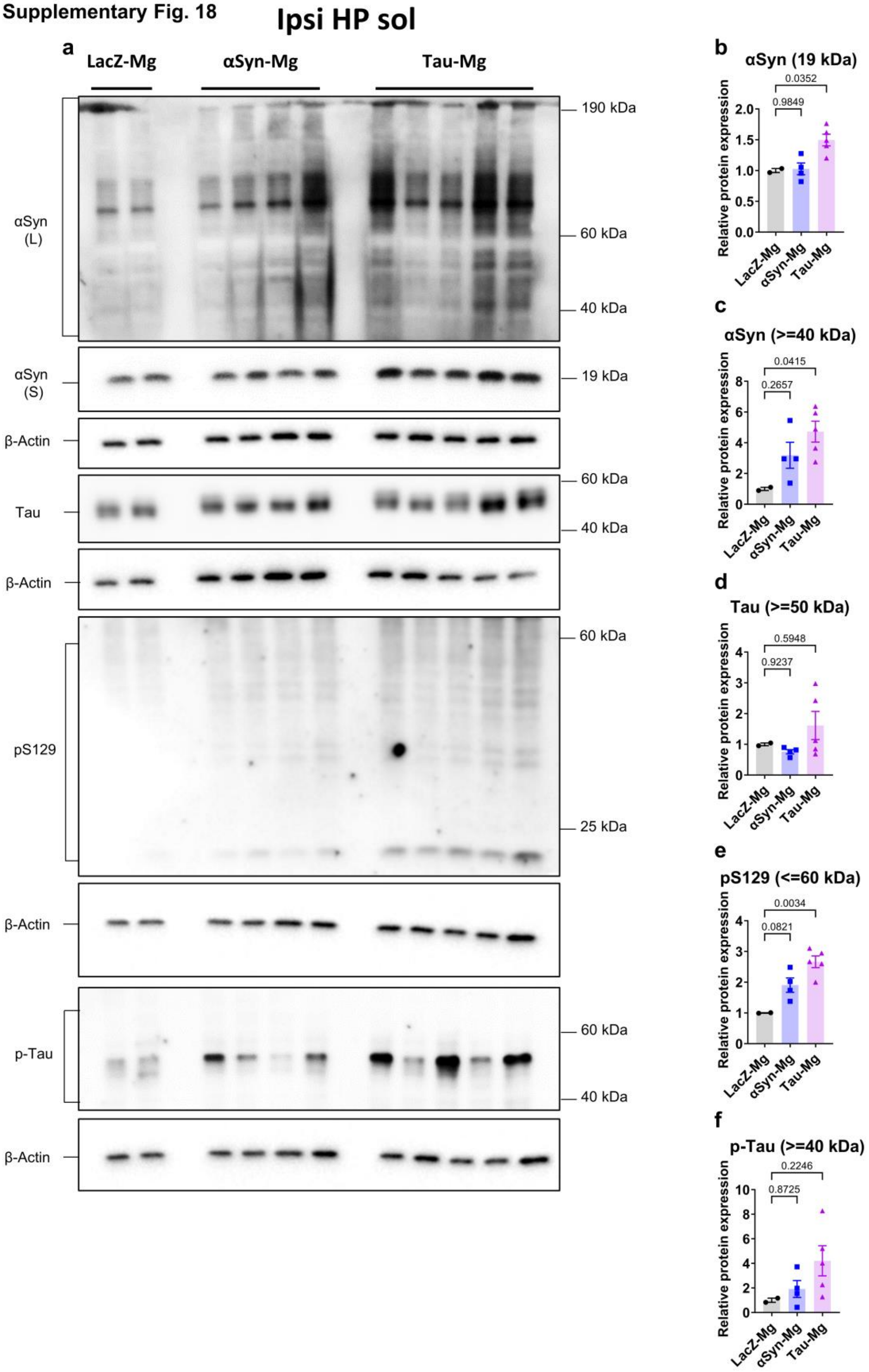

Supplementary Fig. 18: Quantification of the relative protein levels in the Tx-sol fraction of the ipsilateral hippocampus. a Representative western blot images of the Tx-sol fraction of the ipsilateral hippocampus 1 month after injection. L, long exposure time; S, short exposure time. **b-f** The relative expression levels of monomeric α-synuclein (**b**), oligomeric α-synuclein (**c**), Tau (**d**), pS129 (**e**), and p-Tau (**f**). All data are presented as the means ± SEMs. For statistical analysis, one-way ANOVA with Tukey's post hoc test was performed.

Supplementary Fig. 19

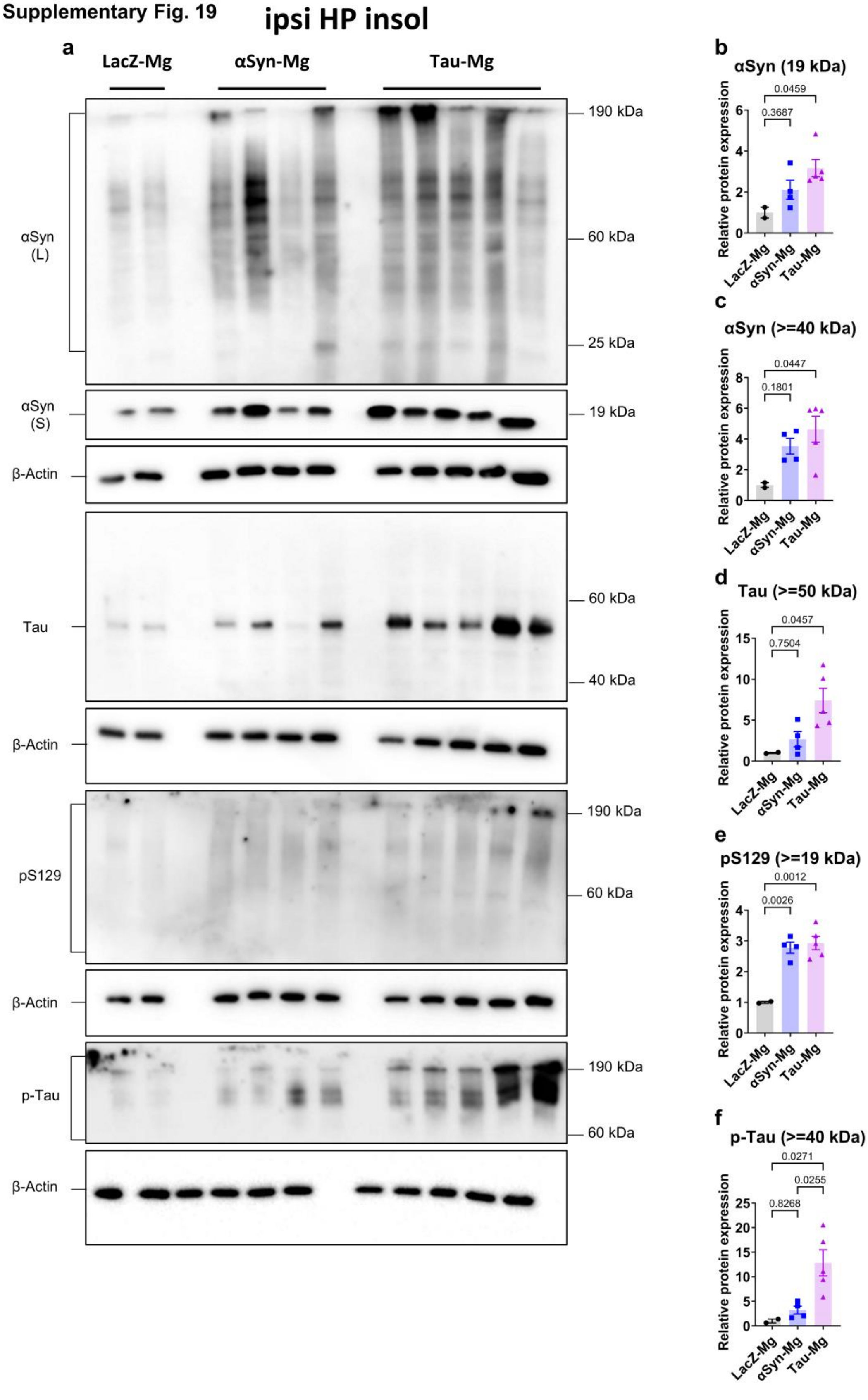

Supplementary Fig. 19: Quantification of the relative protein levels in the Tx-insol fraction of the ipsilateral hippocampus. a Representative western blot images of the Tx-insol fraction of the ipsilateral hippocampus 1 month after injection. L, long exposure time; S, short exposure time. **b-f** The relative expression levels of monomeric α-synuclein (**b**), oligomeric α-synuclein (**c**), Tau (**d**), pS129 (**e**), and p-Tau (**f**). All data are presented as the means ± SEMs. For statistical analysis, one-way ANOVA with Tukey's post hoc test was performed.

Supplementary Fig. 20

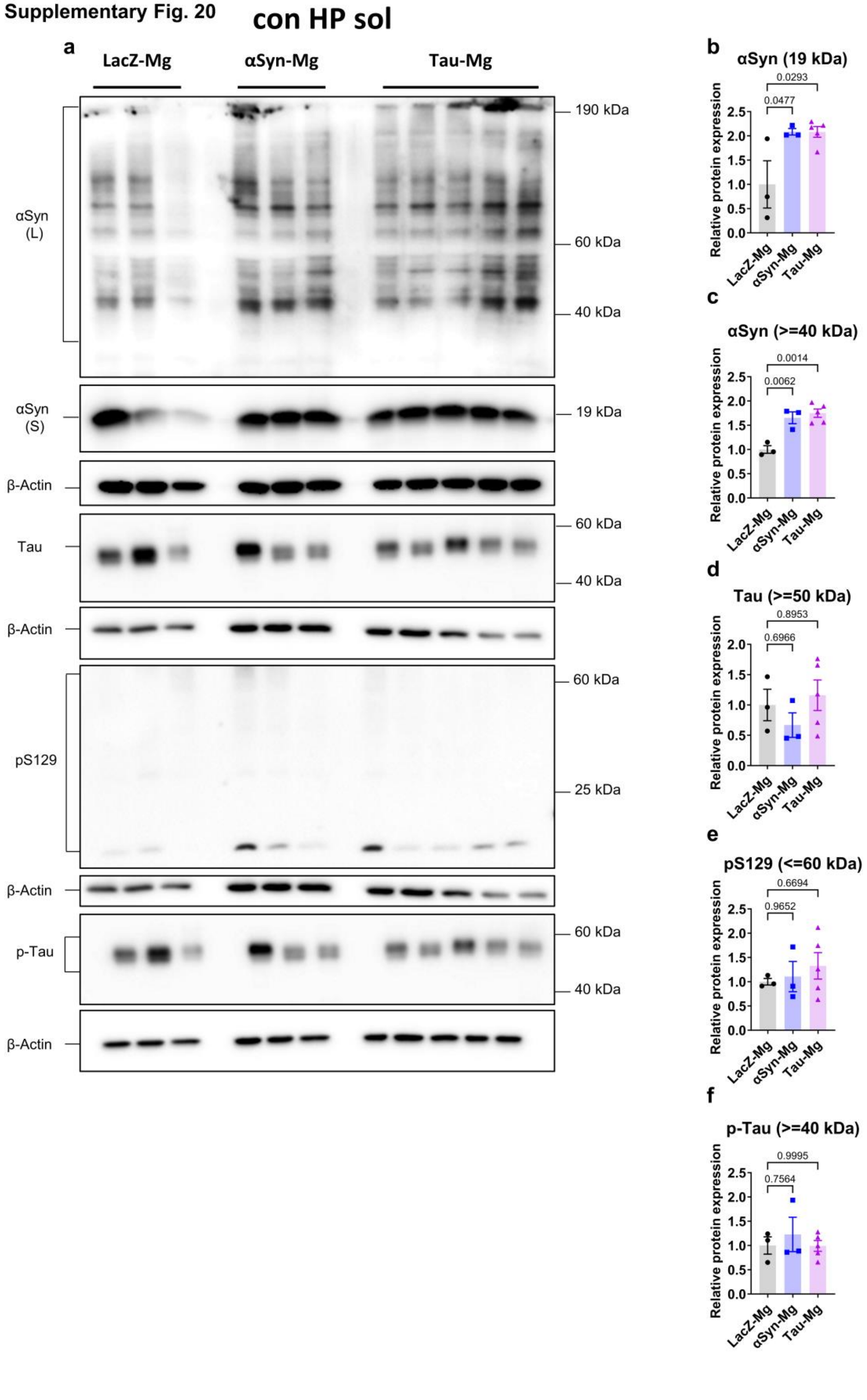

Supplementary Fig. 20: Quantification of the relative protein levels in the Tx-sol fraction of the contralateral hippocampus. a Representative western blot images of the Tx-sol fraction of the contralateral hippocampus 1 month after injection. L, long exposure time; S, short exposure time. **b-f** The relative expression levels of monomeric α-synuclein (**b**), oligomeric α-synuclein (**c**), Tau (**d**), pS129 (**e**), and p-Tau (**f**). All data are presented as the means ± SEMs. For statistical analysis, one-way ANOVA with Tukey's post hoc test was performed.

Supplementary Fig. 21

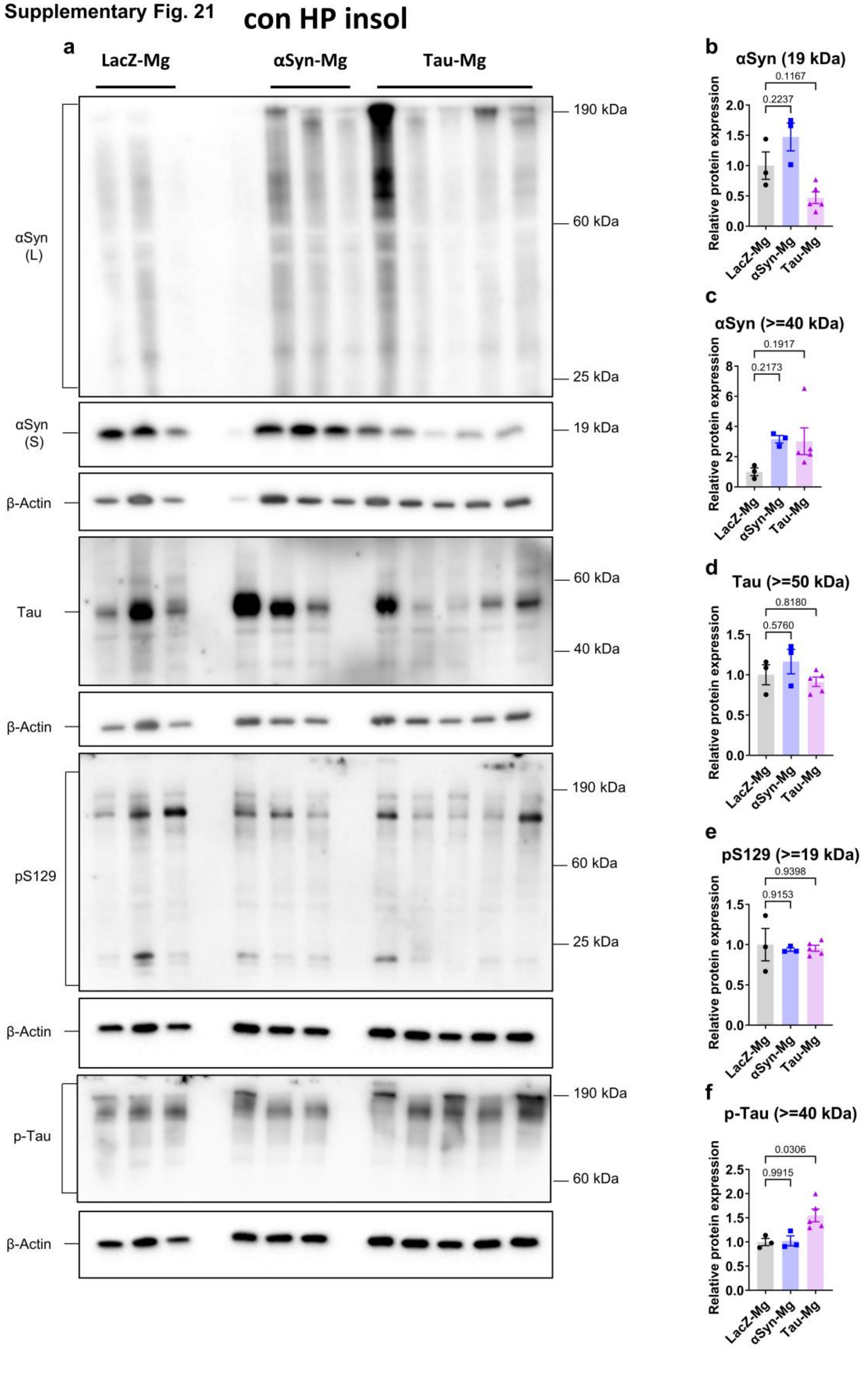

Supplementary Fig. 21: Quantification of the relative protein levels in the Tx-insol fraction of the contralateral hippocampus. a Representative western blot images of the Tx-insol fraction of the contralateral hippocampus 1 month after injection. L, long exposure time; S, short exposure time. **b-f** The relative expression levels of monomeric α-synuclein (**b**), oligomeric α-synuclein (**c**), Tau (**d**), pS129 (**e**), and p-Tau (**f**). All data are presented as the means ± SEMs. For statistical analysis, one-way ANOVA with Tukey's post hoc test was performed.

Supplementary Fig. 22

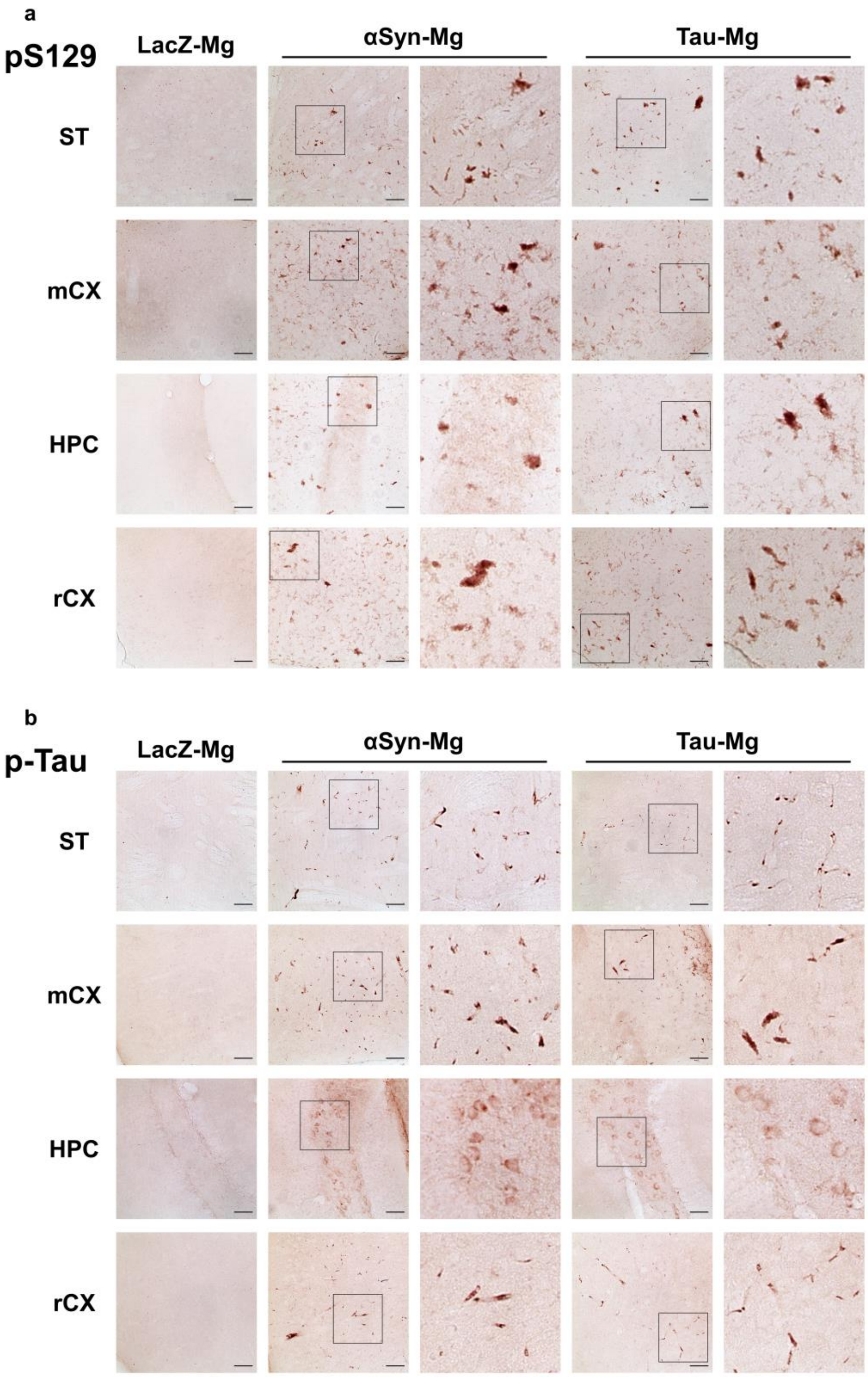

Supplementary Fig. 22: Representative IHC images of the brain regions labeled with pS129 or p-Tau. a-b Representative IHC images of the striatum, motor cortex, hippocampus, and rhinal cortex after labeling with antibodies specific for pS129 (**a**) or p-Tau (**b**) 4 weeks after injection. ROI shown in the black box is magnified. Scale bar, 50 μm.

Supplementary Fig. 23

Substantia nigra

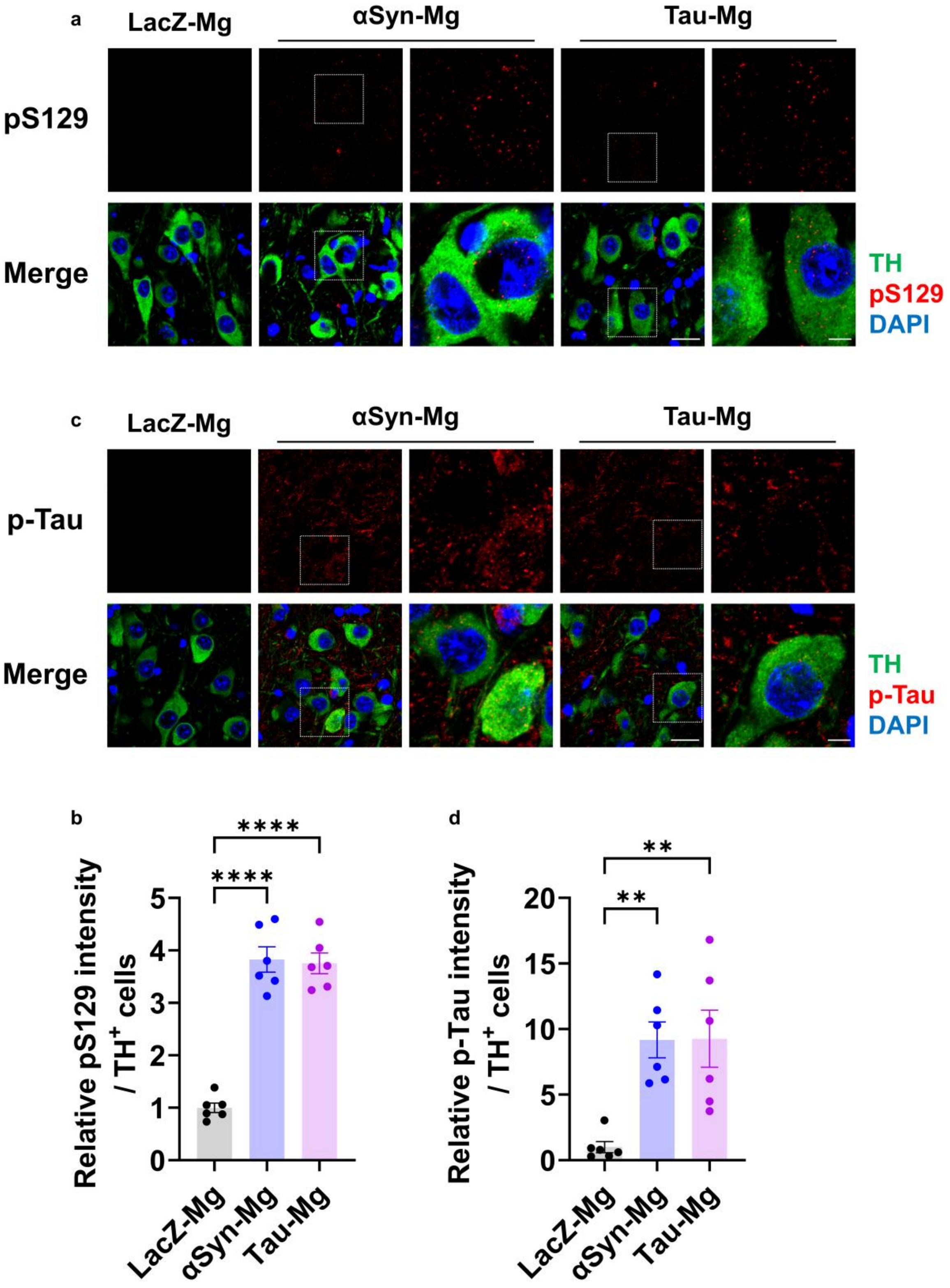

Supplementary Fig. 23: Expression of pS129 and p-Tau in the substantia nigra 4 weeks post-injection. a-b Representative IF images of the substantia nigra costained with TH/pS129 (**a**) and the corresponding relative pS129 levels per TH-positive cell (**b**). **c-d** Representative IF images of the substantia nigra costained with TH/p-Tau (**c**) and the corresponding relative p-Tau levels per TH-positive cell (**d**). For **b** and **d**, values were normalized to the LacZ-Mg group. All data are presented as the means ± SEMs. For statistical analysis, one-way ANOVA with Tukey's post hoc test was performed. For **a** and **c**, the ROI outlined in the white box is magnified. Scale bar, 20 μm; 5 μm for magnified images.

Supplementary Fig. 24

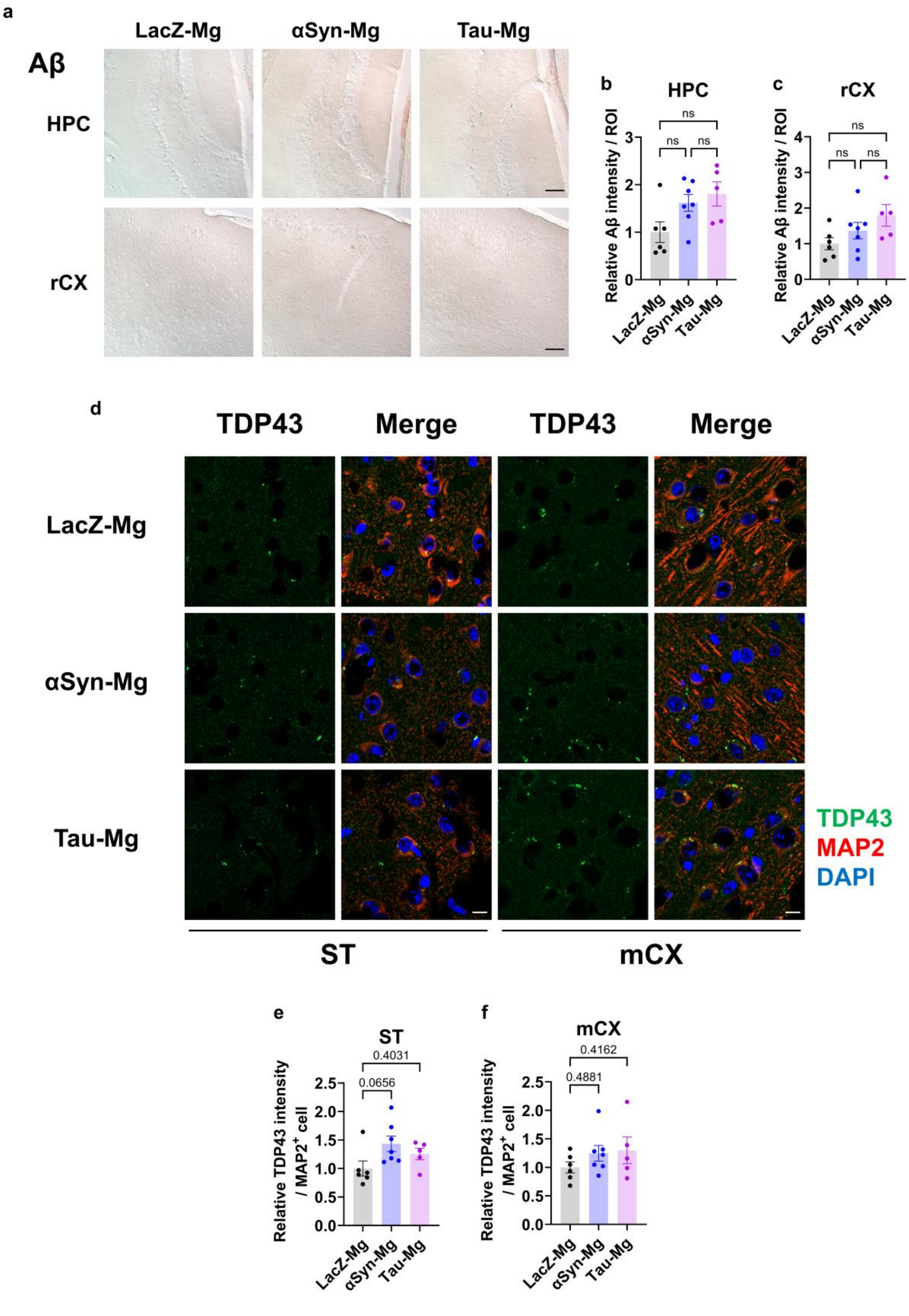

Supplementary Fig. 24: Expression of Aβ and TDP43 4 weeks post-injection. a-c Representative IHC images of the HPC and rCX labeled with an antibody specific for Aβ (**a**) and relative Aβ levels in the HPC (**b**) and rCX (**c**). Scale bar, 50 μm. **d-f** Representative IF images of the ST and mCX labeled with an antibody specific for TDP43 (**d**) and relative neuronal TDP43 levels in the ST (**e**) and mCX (**f**). For **b-c** and **e-f**, values from the ipsilateral and contralateral hemispheres were averaged and normalized to those of the LacZ-Mg group. All data are presented as the means ± SEMs. For statistical analysis, one-way ANOVA with Tukey's post hoc test was performed.

Supplementary Fig. 25

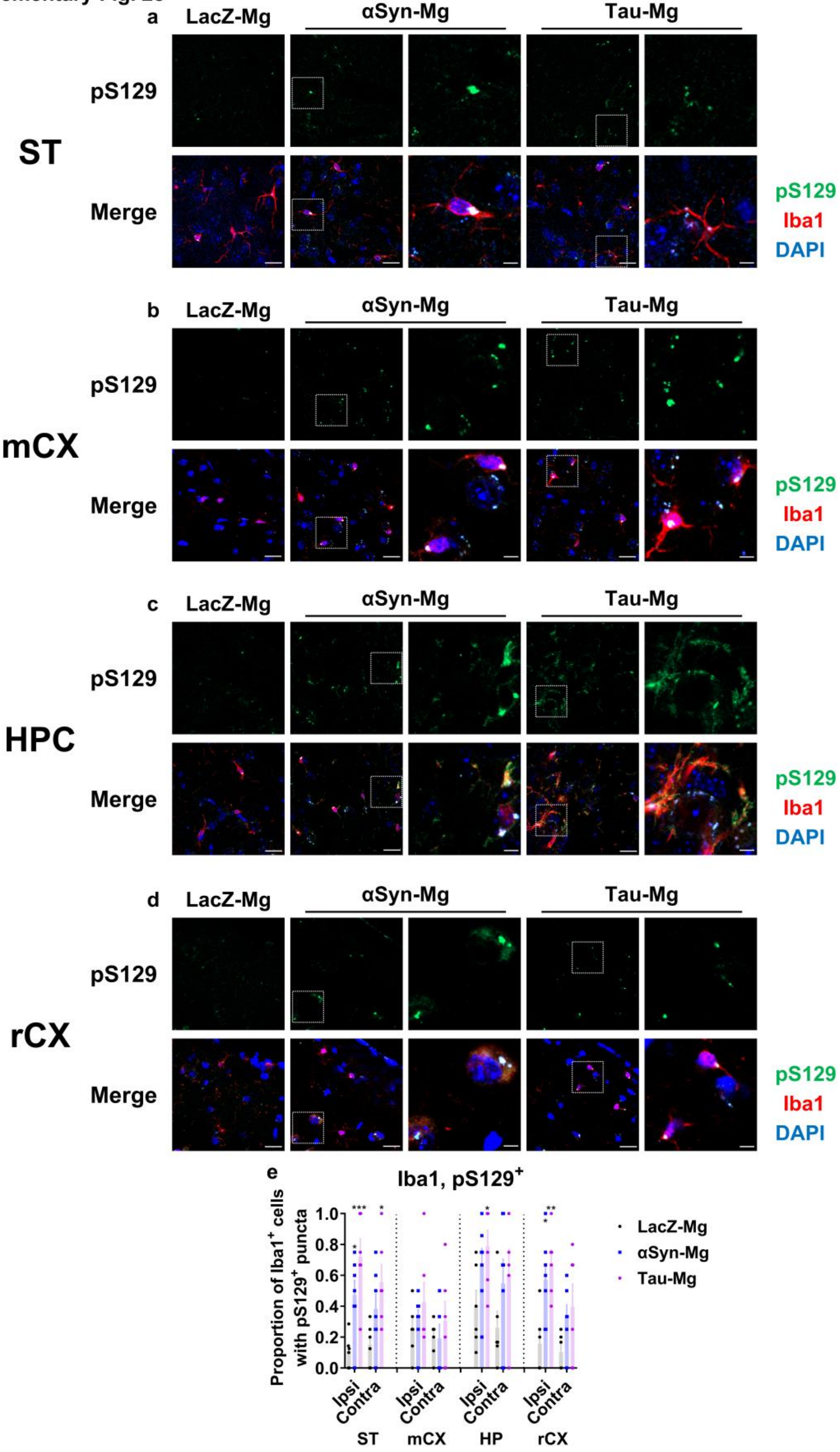

Supplementary Fig. 25: Costaining of the brain regions with Iba1 and pS129. a-d Representative IF images costained with Iba1 and pS129 in the ipsilateral striatum (**a**), motor cortex (**b**), hippocampus (**c**), and rhinal cortex (**d**) 1 month after injection. ROI shown in the white box is magnified. Scale bar, 20 μm; and 10 μm for magnified images. **e** Proportion of $Iba1^+$ cells with $pS129^+$ inclusions. All data are presented as the means ± SEMs. For statistical analysis, two-way ANOVA followed by Tukey's post hoc test was performed.

Supplementary Fig. 26

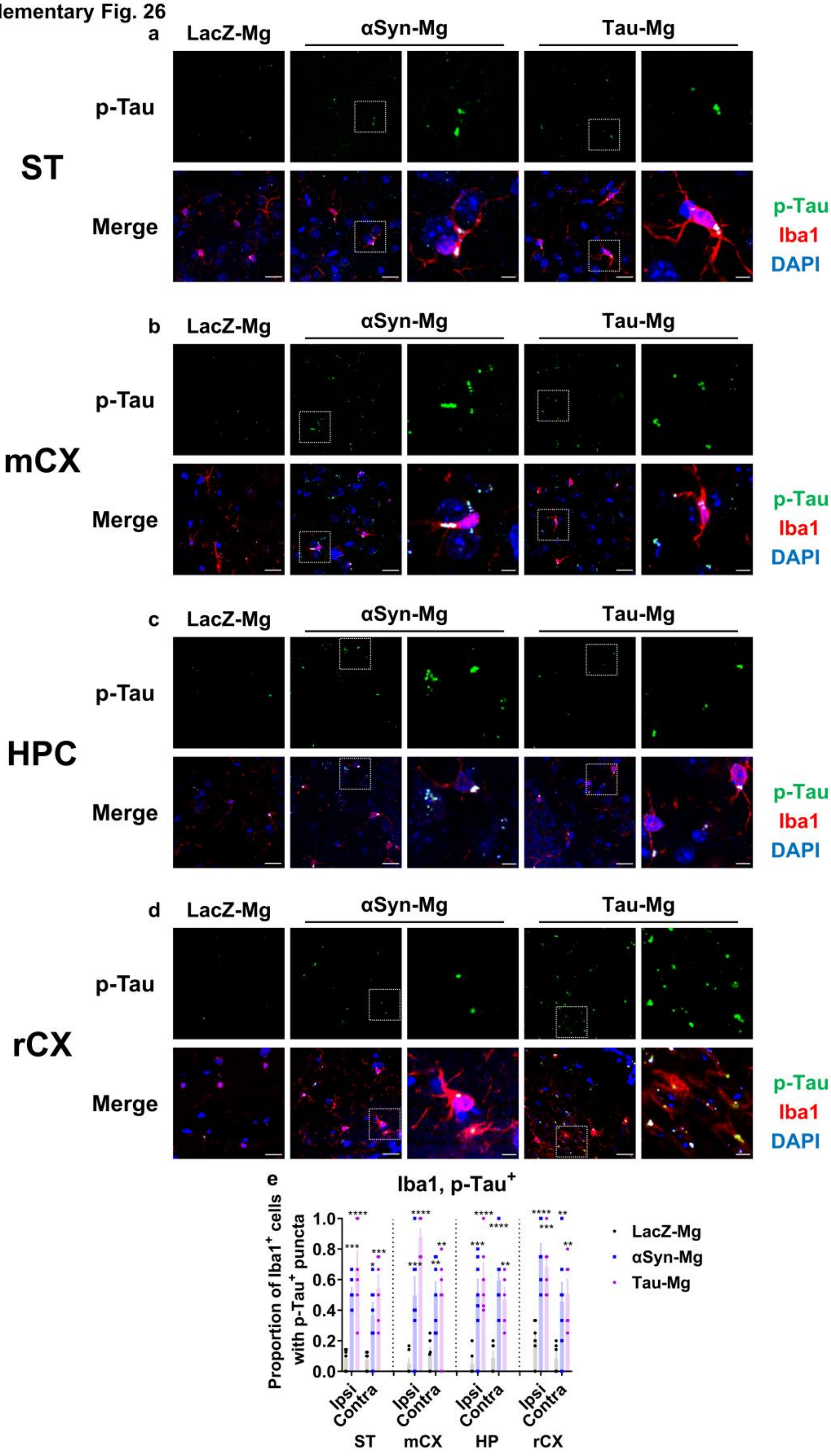

Supplementary Fig. 26: Costaining of the brain regions with Iba1 and p-Tau. a-d Representative IF images costained with Iba1 and p-Tau in the ipsilateral striatum (**a**), motor cortex (**b**), hippocampus (**c**), and rhinal cortex (**d**) 1 month after injection. ROI shown in the white box is magnified. Scale bar, 20 μm; and 10 μm for magnified images. **e** Proportion of Iba1$^+$ cells with p-Tau$^+$ inclusions. All data are presented as the means ± SEMs. For statistical analysis, two-way ANOVA followed by Tukey's post hoc test was performed.

Supplementary Fig. 27

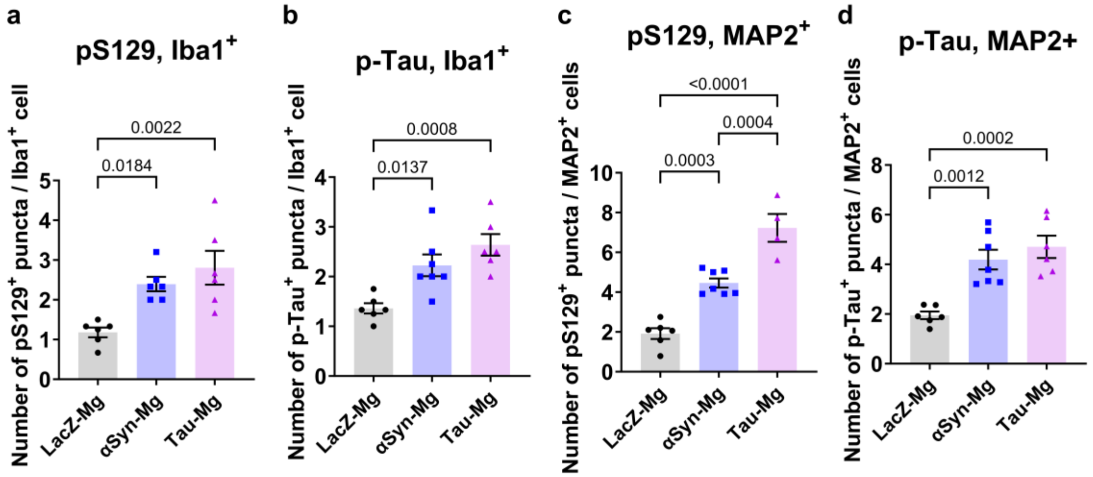

Supplementary Fig. 27: The number of pS129 and p-Tau puncta in neurons and microglia. **a-d** The number of pS129-positive inclusions per Iba1-positive cell (**a**), p-Tau-positive inclusions per Iba1-positive cell (**b**), pS129-positive inclusions per MAP2-positive cell (**c**), and p-Tau-positive inclusions per MAP2-positive cell (**d**) in the ipsilateral motor cortex 1 month after injection. All data are presented as the means ± SEMs. For statistical analysis, one-way ANOVA with Tukey's post hoc test was performed.

Supplementary Fig. 28

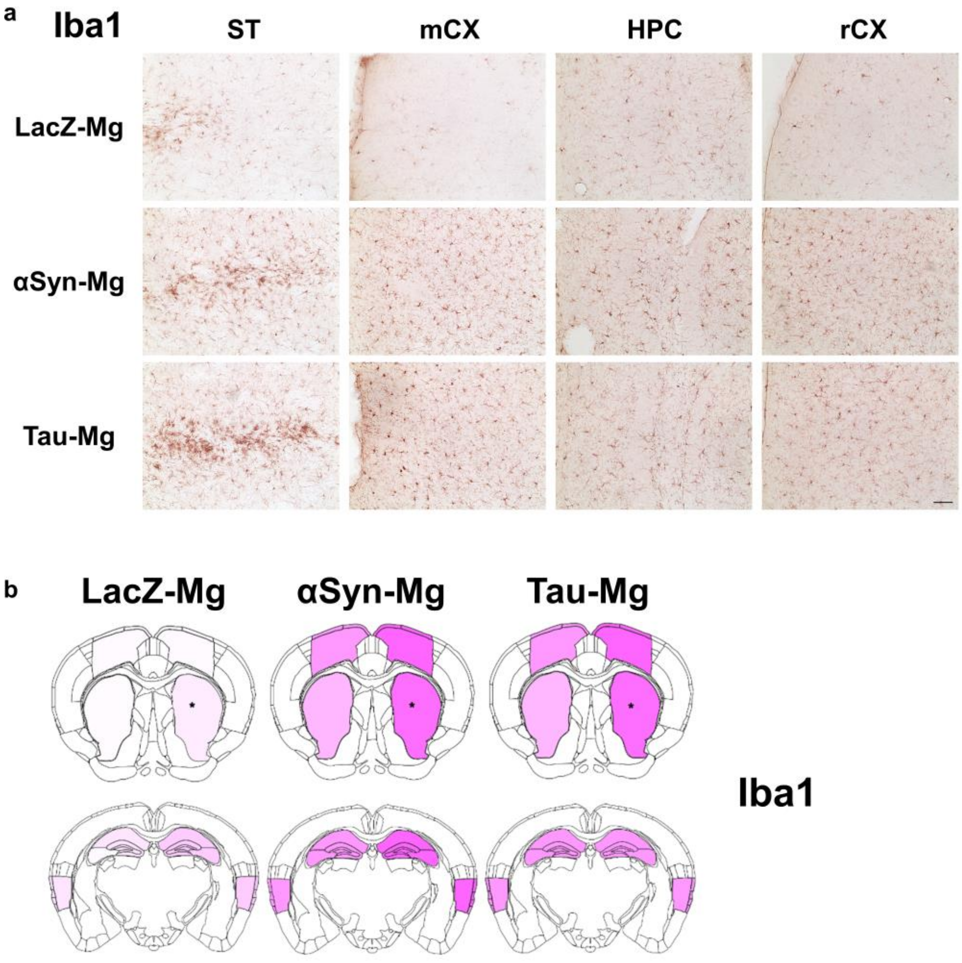

Supplementary Fig. 28: Representative IHC images of the brain regions labeled with Iba1. **a** Representative IHC images of the striatum, motor cortex, hippocampus, and rhinal cortex after labeling with antibodies specific for Iba1 1 month after injection. Scale bar, 100 µm. **b** Heatmaps depicting the expression patterns of Iba1. Asterisks mark the injection sites.

Supplementary Fig. 29

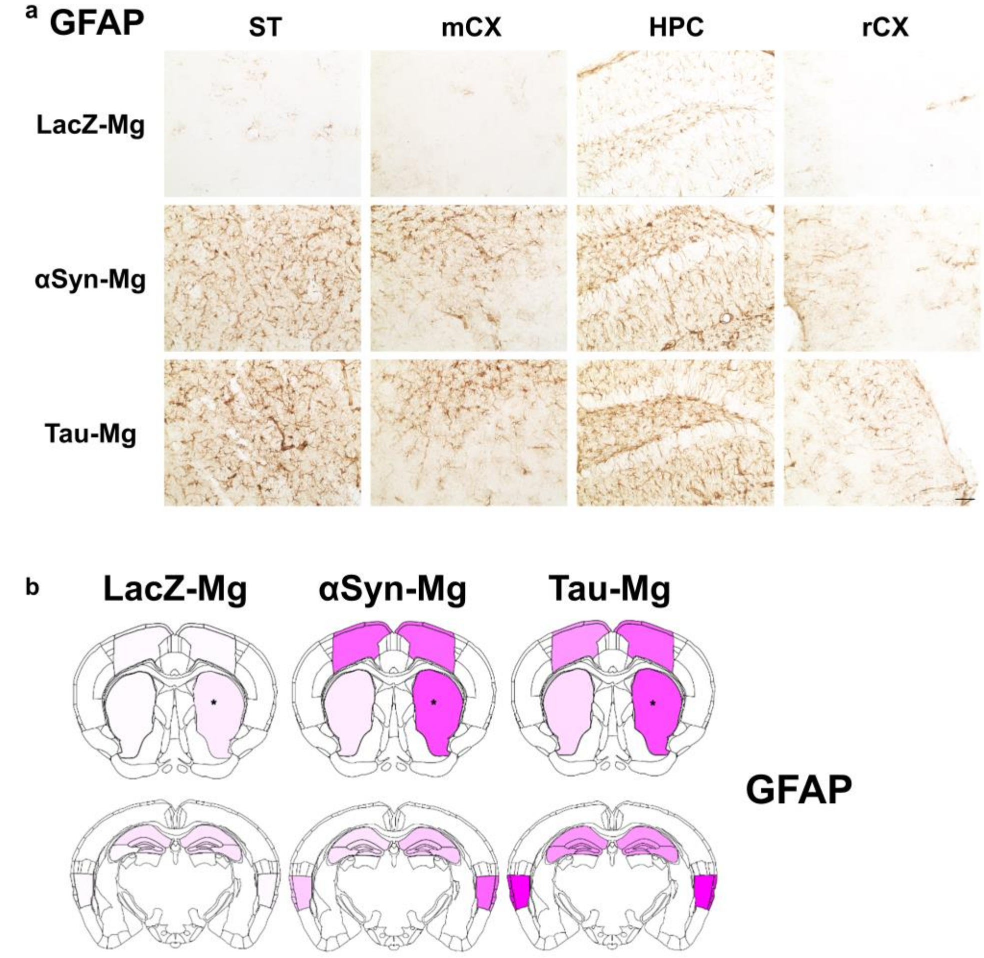

Supplementary Fig. 29: Representative IHC images of the brain regions labeled with GFAP. a Representative IHC images of the striatum, motor cortex, hippocampus, and rhinal cortex after labeling with antibodies specific for GFAP 1 month after injection. Scale bar, 100 μm. **b** Heatmaps depicting the expression patterns of GFAP. Asterisks mark the injection sites.

Supplementary Fig. 30

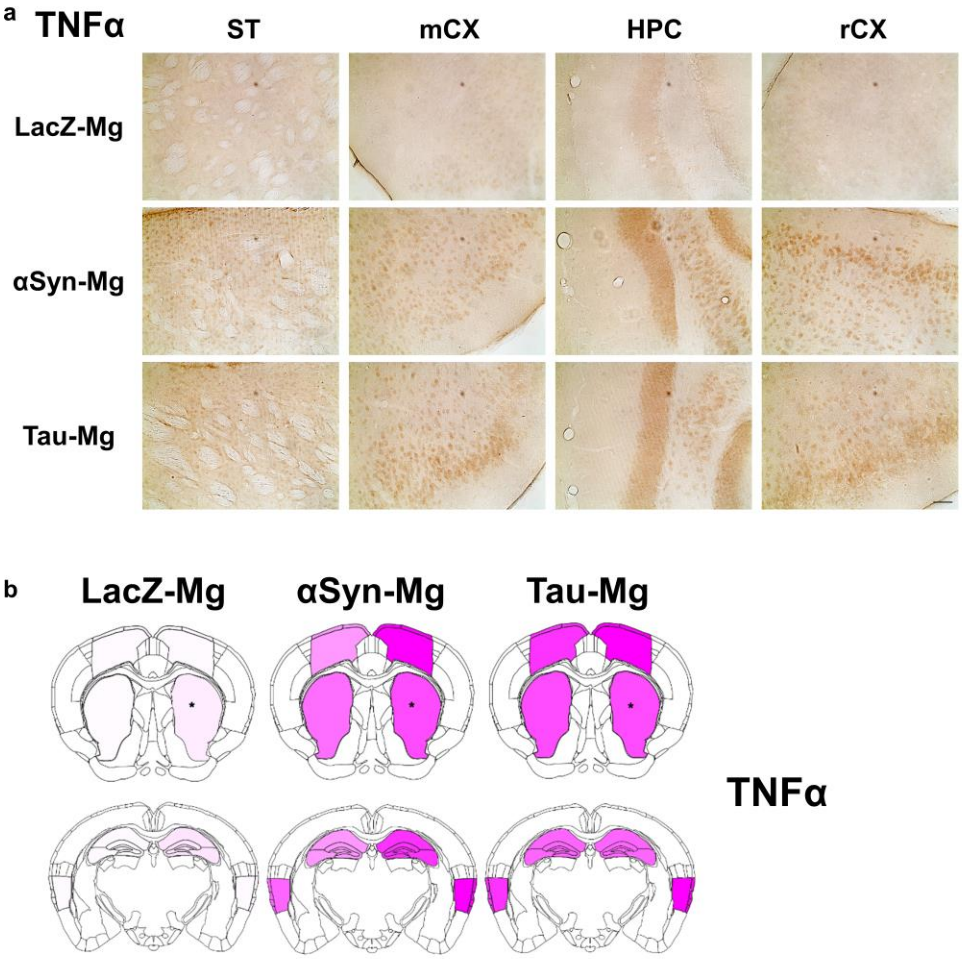

Supplementary Fig. 30: Representative IHC images of the brain regions labeled with TNFα. a Representative IHC images of the striatum, motor cortex, hippocampus, and rhinal cortex after labeling with antibodies specific for TNFα 1 month after injection. Scale bar, 100 μm. **b** Heatmaps depicting the expression patterns of TNFα. Asterisks mark the injection sites.

Supplementary Fig. 31

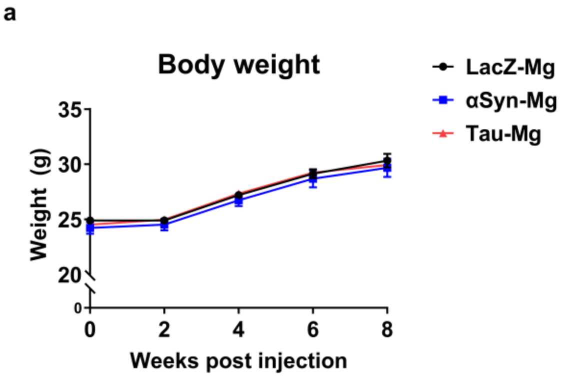

Supplementary Fig. 31: The body weight of mice injected with activated microglia. a The body weight of mice after transplantation of activated microglia into the striatum. All data are presented as the means ± SEMs. For statistical analysis, two-way repeated measures ANOVA followed by Sidak's post hoc test was performed.
